# SAGA mediates transcription from the TATA-like element independently of Taf1p/TFIID but dependent on core promoter structures in *Saccharomyces cerevisiae*

**DOI:** 10.1371/journal.pone.0188435

**Published:** 2017-11-27

**Authors:** Kiyoshi Watanabe, Tetsuro Kokubo

**Affiliations:** Molecular and Cellular Biology Laboratory, Graduate School of Medical Life Science, Yokohama City University, Yokohama, Kanagawa, Japan; Institute of Genetics and Molecular and Cellular Biology, FRANCE

## Abstract

In *Saccharomyces cerevisiae*, core promoters of class II genes contain a TATA element, either a TATA box (TATA[A/T]A[A/T][A/G]) or TATA-like element (1 or 2 bp mismatched version of the TATA box). The TATA element directs the assembly of the preinitiation complex (PIC) to ensure accurate transcriptional initiation. It has been proposed the PIC is assembled by two distinct pathways in which TBP is delivered by TFIID or SAGA, leading to the widely accepted model that these complexes mediate transcription mainly from TATA-like element- or TATA box-containing promoters, respectively. Although both complexes are involved in transcription of nearly all class II genes, it remains unclear how efficiently SAGA mediates transcription from TATA-like element-containing promoters independently of TFIID. We found that transcription from the TATA box-containing *AGP1* promoter was greatly stimulated in a Spt3p-dependent manner after inactivation of Taf1p/TFIID. Thus, this promoter provides a novel experimental system in which to evaluate SAGA-mediated transcription from TATA-like element(s). We quantitatively measured transcription from various TATA-like elements in the Taf1p-dependent *CYC1* promoter and Taf1p-independent *AGP1* promoter. The results revealed that SAGA could mediate transcription from at least some TATA-like elements independently of Taf1p/TFIID, and that Taf1p-dependence or -independence is highly robust with respect to variation of the TATA sequence. Furthermore, chimeric promoter mapping revealed that Taf1p-dependence or independence was conferred by the upstream activating sequence (UAS), whereas Spt3p-dependent transcriptional stimulation after inactivation of Taf1p/TFIID was specific to the *AGP1* promoter and dependent on core promoter regions other than the TATA box. These results suggest that TFIID and/or SAGA are regulated in two steps: the UAS first specifies TFIID or SAGA as the predominant factor on a given promoter, and then the core promoter structure guides the pertinent factor to conduct transcription in an appropriate manner.

## Introduction

In eukaryotes, general transcription factors (GTFs), Mediator, and RNA polymerase II (pol II) assemble on the core promoter to form a preinitiation complex (PIC) that directs accurate transcriptional initiation [1–5]. In the first step of PIC assembly, TBP is recruited to the core promoter as a subunit of TFIID or via physical association with the SPT module of the SAGA (Spt-Ada-Gcn5-acetyltransferase) complex [6–9]. TFIID and SAGA are structurally related large multi-protein complexes that mediate basal and/or activated transcription [2, 10–12]. They share five Taf subunits that form a scaffold for the assembly of other complex-specific subunits [9, 13–16], and the two complexes define two distinct PIC assembly pathways [4, 8, 17].

Genome-wide studies revealed that SAGA-dominated promoters (i.e., those whose transcriptional activities are primarily supported by SAGA rather than by TFIID) prefer the TATA box (TATAWAWR; W = A/T, R = A/G), whereas TFIID-dominated promoters prefer the TATA-like element (a 1 or 2 bp mismatched version of the TATA box) [7, 8]. According to the nomenclature proposed by Rhee and Pugh [8], the TATA element includes the TATA box and the TATA-like element. More recent studies indicate that TFIID and SAGA are both involved in transcription of nearly all class II genes [18–21]. Consistent with this, it is well established that TFIID can mediate transcription from various types of promoters, both *in vivo* and *in vitro*, regardless of whether they contain the TATA box or not [22–28]. However, it remains unclear how efficiently SAGA can mediate transcription from TATA-less promoters that do not contain the TATA box but instead contain TATA-like elements or other less well-characterized core promoter elements (CEs) [25, 29, 30], especially in a manner independent of TFIID.

One reason for this ambiguity is the scarcity of *in vitro* experiments that scrutinize the requirements of the TATA sequence for SAGA-dependent transcription. In an *in vitro* SAGA-dependent transcription system, SAGA can mediate transcription from the TATA-containing *HIS4* promoter [31] but not from the TATA-less *RPS5* promoter [24]. However, it remains to be determined whether SAGA can mediate transcription from a *HIS4* promoter containing a TATA-like element (off-consensus TATA) or a *RPS5* promoter containing a consensus TATA box under the same conditions. Other studies showed that several sequences isolated as active CEs in a random screen using the *gal-his3* hybrid promoter [32] have significant transcriptional activities *in vitro*, even if they do not contain a consensus TATA box [33], but it remains unclear whether the *in vitro* transcription system used in the latter experiment is SAGA-dependent.

Another reason for the ambiguity is the functional redundancy between TFIID and SAGA [7, 34]. In general, SAGA-dependent promoters are highly regulated [7, 35] and require the TATA box for transcriptional activation [35, 36], as observed for the *GAL1* promoter [37–40]. Consistent with this, mutational studies of the partially (i.e., subunit-specifically) SAGA-dependent [11] but TFIID-independent [41, 42] T_R_ element of the *HIS3* promoter revealed a rather stringent requirement of the TATA sequence for transcription [43, 44]. However, the TATA-less *TRP3* promoter [45] is regulated not only by TFIID [7, 41, 42] but also by SAGA in a manner very similar to the *HIS3-T*_*R*_ promoter [11]. Based on these findings, it is certain that TFIID plays an essential role in transcription from the TATA-less *TRP3* promoter, but not from the *HIS3-T*_*R*_ promoter. However, these observations imply that, in a SAGA-defective strain, TFIID could alter its role in transcription to generate similar transcriptional profiles for these two promoters. If this is the case, it will be important to carefully evaluate how efficiently SAGA mediates transcription from TATA-less promoters under a condition in which the contribution of TFIID to transcription is minimized, e.g., by functional inactivation of TFIID-specific Taf(s).

Recent studies suggest that transcriptional attenuation, e.g., due to a malfunction of the transcriptional machinery, could be compensated by down-regulation of mRNA degradation [18, 20, 21, 46–49]. Presumably due to such a buffering effect, only limited genome-wide defects can be observed when steady-state mRNA levels are measured in TFIID- or SAGA-defective strains [7, 50]. In fact, measurements of nascent mRNA levels revealed that TFIID and SAGA are both globally required for transcription of nearly all class II genes, regardless of whether they contain the TATA box [20, 21]. These results indicate that the observed defects in the production of steady-state mRNA may not result from reduced transcription per se but rather from the combined effects of a decrease in transcription and an increase in mRNA stabilization. Therefore, TFIID- and SAGA-dominated promoters cannot be distinguished simply by the transcriptional requirements for these two factors [20, 21]. Despite these recent advances, it remains unclear how TFIID and SAGA mediate transcription from the two types of promoters.

Previously [51], we randomized the TATAα element in the Taf1p/TFIID-dependent *CYC1* promoter to search for transcriptionally active CEs. The active 601 sequences (from 4,781 clones) obtained in that screen were tentatively classified into nine groups: classes I (TATAWAWR; consensus TATA box), II (TATAWAD; D = A/G/T), III (GAAAA), IV (TATAWKW; K = G/T), V (TTAAAW), VI (Wx6), VII (TATATCWD), VIII (Wx5 other than TATATCWD), and IX (others) [51]. Consistent with the findings of genome-wide studies [7, 8], we found that the Taf1p/TFIID-dependent *CYC1* promoter could use various CE sequences that did not match the consensus TATA box [51].

In this study, to determine how efficiently SAGA mediates transcription from TATA-less promoters independently of Taf1p/TFIID, we examined the transcriptional activities of several CE sequences belonging to class I, II, V, or VI in the Taf1p/TFIID-independent *AGP1* promoter under conditions in which Taf1p was functionally inactivated by temperature shift and residual transcription was entirely Spt3p/SAGA-dependent. Furthermore, we also mapped the determinant(s) of Taf1p/TFIID-dependence and -independence of the *CYC1* or *AGP1* promoters, respectively, as well as those of Spt3p/SAGA-dependent transcriptional stimulation of the *AGP1* promoter after inactivation of Taf1p/TFIID. The results indicated that the function of TFIID and/or SAGA is regulated in two steps, in which the upstream activating sequence (UAS) and core promoter play specific roles and probably operate in a sequential manner.

## Materials and methods

### Yeast strains

Standard techniques were used for yeast growth and transformation [52]. Yeast strains used in this study are listed in S1 Table. Sequences of oligonucleotides used for strain or plasmid construction are listed in S2 Table.

All strains used in this study, except those depicted in S1 and S3 Figs, were derived from Y22.1, which carries a deletion of the chromosomal *TAF1* coding region and the wild-type *TAF1* gene in a *URA3*-based low-copy-number vector (pYN1) [53]. YTK2741 [54] and YTK3778 [51] were generated from Y22.1 by replacing pYN1 with pM1169 (HA-tagged wild-type *TAF1*/pRS314) [55] and pM1746 (HA-tagged *taf1-N568Δ*/pRS314) [51], respectively. BY4741 and Y04228 were obtained from Euroscarf, and YTK16396/16397/16398/16399 were described previously [51].

To create YTK17952 and YTK17974, the two sub-fragments containing *AGP1* [-780 – -111 bp] (primers: TK8267-TK8678/template: genomic DNA of BY4741; hereafter abbreviated as TK8267-TK8678/BY4741) or *AGP1* [-135 – -1 bp] (TK12517-TK8268/BY4741) were first amplified by PCR using the primer pair/template (genomic DNA or plasmid) as described above in parenthesis, and then fused with TK8267-TK8268 to generate a 0.84 kb fragment (S3 Table). The original TATA box (TATATAAG) of the *AGP1* promoter was replaced with a different one (TATATAAA) derived from the *CYC1* promoter. Subsequently, the other two sub-fragments containing *VTC1* [-320 – -11 bp] + *LEU2* [56] (TK7872-TK6582/YTK16396) or *VTC1* [+1 – +220 bp] (TK9030-TK9171/BY4741) were amplified by PCR. Finally, these two sub-fragments and the aforementioned 0.84 kb fragment were fused with TK7872-TK9171 to generate a 3.6 kb fragment that was used for transformation of YTK2741 and YTK3778, yielding YTK17952 and YTK17974, respectively (S3 Table). Similar to YTK17952, YTK17954/17964/17966/17968/17970/17956/17958/17960/17962/17972/17586/17584/17591/17593/17582 were generated from YTK2741 by transforming PCR fragments amplified using primers TK12518/13002/12524/12525/12526/12519/12520/12521/ 12522/12527/12926/12925/12928/12929/12924, respectively, instead of TK12517 (S3 Table). Furthermore, similar to YTK17974, YTK17976/17978/17980/17982/17984/ 17599/17597/17604/17606/17595 were generated from YTK3778 by transforming PCR fragments amplified using primers TK12518/12519/12520/12521/12522/12926/ 12925/12928/12929/12924, respectively, instead of TK12517 (S3 Table).

To create YTK17590 and YTK17603, the three sub-fragments containing *VTC1* [-320 – -11 bp] + *LEU2* [56] (TK7872-TK6582/YTK16396), *AGP1* [-780 – -1 bp] (TK8267-TK8268/BY4741), or *VTC1* [+1 – +220 bp] (TK9030-TK9171/BY4741) were first amplified by PCR, and then fused with TK7872-TK9171 to generate a 3.6 kb fragment that was used for transformation of YTK2741 and YTK3778, yielding YTK17590 and YTK17603, respectively (S3 Table).

To create YTK16408/16410/16412/16414/16400/16402/16404/16406/16416 and YTK16401/16403/16405/16407, the three sub-fragments containing *VTC1* [-530 – -11 bp] (TK10267-TK7873/BY4741), *LEU2* (TK12262-TK6582/pUG73 [56]), or the *CYC1* promoter [-400 – -1 bp] + *VTC1* [+1 – +430 bp] (TK10036-TK4283/genomic DNA derived from each strain that had been isolated by a previous screen [51]) were first amplified by PCR, and then fused by TK10267-TK4283 to generate a 3.6 kb fragment that was used for transformation of YTK2741 and YTK3778, respectively (S3 Table).

To create YTK17566 and YTK17578, the two sub-fragments containing *VTC1* [-400 – -11 bp] + *LEU2* marker [56] + the *CYC1* promoter [-400 – -124 bp] (TK8260-TK10257/YTK16396), or the *CYC1* promoter [-123 – -1 bp] + *VTC1* [+1 – +320 bp] (TK12922-TK7875/YTK16396), were first amplified by PCR, and then fused with TK8260-TK7875 to generate a 3.4 kb fragment that was used for transformation of YTK2741 and YTK3778, respectively (S3 Table). Similar to YTK17566, YTK17562/17560/17564/17568/17558 were generated from YTK2741 by transforming PCR fragments amplified using primers TK12920/12919/12921/12923/ 12918, respectively, instead of TK12922 (S3 Table). Furthermore, similar to YTK17578, YTK17574/17572/17576/17580/17570 were generated from YTK3778 by transforming PCR fragments amplified using primers TK12920/12919/12921/12923/12918, respectively, instead of TK12922 (S3 Table).

To create YTK17748 and YTK17784, the fragments containing *VTC1* [-170 – -11 bp] + *LEU2* [56] + *AGP1*_*UAS*_ [-780 – -301 bp] + *CYC1*_*core*_ [-230 – -1 bp] + *VTC1* [+1 – +150 bp] were amplified by PCR (TK2496-TK10408/pM8010 carrying *AGP1*_*UAS*_*+CYC1*_*core*_ [TATATAAA]) to generate a 3.3 kb fragment that was used for transformation of YTK2741 and YTK3778, respectively. Similarly, YTK17750/17766/17769 and YTK17786/17802/17804 were generated from YTK2741 and YTK3778, respectively, using pM8011 (*AGP1*_*UAS*_*+CYC1*_*core*_ [TAGCGCAA])/ pM8019 (*CYC1*_*UAS*_*+AGP1*_*core*_ [TATATAAA])/ pM8020 (*CYC1*_*UAS*_*+ AGP1*_*core*_ [TAGCGCAA]) as a template instead of pM8010.

The strains used in S1 and S3 Figs were derived from BY4741 or BY4742. YTK11411, YTK11705, and YTK11708 were described previously [57]. YTK11705 (*TAF1*) and Y04228 (*Δspt3*) were crossed and dissected to generate YTK11871 (*TAF1 spt3*). Similarly, YTK11708 (*taf1-N568Δ*) and Y04228 (*Δspt3*) were crossed and dissected to generate YTK11873 (*taf1-N568Δ Δspt3*). YTK13039 was generated from YTK11871 by replacing *HIS3*-marked pM4770/*TAF1* with *URA3*-marked pYN1/*TAF1*. To create YTK18955 and YTK18964, the fragments containing *VTC1* [-170 – -11 bp] + *LEU2* [56] + *AGP1* promoter [-780 – +1 bp] + *VTC1* [+1 – +150 bp] were amplified by PCR (TK2496-TK10408/YTK17952) to generate a 3.3 kb fragment that was used for transformation of YTK11411 and YTK13039, respectively. YTK18974 and YTK18986 were generated from YTK18955 by replacing pYN1/*TAF1* with pM4770/*TAF1* and pM4773/*taf1-N568Δ*, respectively. Similarly, YTK18998 and YTK19010 were generated from YTK18964 by replacing pYN1/*TAF1* with pM4770/*TAF1* and pM4773/*taf1-N568Δ*, respectively.

### Construction of plasmids

pYN1, pM4770, and pM4773 were described previously [53, 57]. To create pM8010 containing *AGP1*_*UAS*_*+CYC1*_*core*_ [TATATAAA], the two sub-fragments containing *VTC1* [-320 – -11 bp] + *LEU2* [56] + *AGP1*_*UAS*_ [-780 – -301 bp] (TK13578-TK13612/YTK17590), or *CYC1*_*core*_ [-230 – -1 bp] + *VTC1* [+1 – +814 bp] (TK13619-TK13579/YTK16396), and a linearized vector (TK12873-TK13577/pBluescript II KS+) were first amplified by PCR, and then ligated together using the In-Fusion HD cloning kit (TaKaRa). pM8011 containing *AGP1*_*UAS*_*+CYC1*_*core*_ [TAGCGCAA] was created in the same way, except that YTK16398 was used as a template instead of YTK16396.

To create pM8019 containing *CYC1*_*UAS*_*+AGP1*_*core*_ [TATATAAA], the two sub-fragments containing *VTC1* [-320 – -11 bp] + *LEU2* [56] + *CYC1*_*UAS*_ [-400 – -231 bp] (TK13578-TK13618/YTK16396), or *AGP1*_*core*_ [-300 – -1 bp] + *VTC1* [+1 – +814 bp] (TK13614-TK13579/YTK17952), and a linearized vector (TK12873-TK13577/pBluescript II KS+) were first amplified by PCR, and then ligated together using the In-Fusion HD cloning kit. pM8020 containing *CYC1*_*UAS*_*+AGP1*_*core*_ [TAGCGCAA] was created in the same way, except that YTK17954 was used as a template instead of YTK17952. These plasmids (i.e., pM8010/8011/8019/8020) were used as PCR templates for the construction of eight yeast strains (i.e., YTK17748/17750/17766/17769/17784/17786/7802/17804) as described above.

### Northern blot analysis

Northern blot analysis was performed as described previously [58]. Specifically, total RNA (20 μg) was isolated from the indicated strains grown to logarithmic phase at 25°C in YPD media or further incubated at 37°C for the indicated period of time. RNA was subjected to electrophoresis, blotted onto a membrane, and hybridized with the gene-specific probes.

For detection of *VTC1*, *CYC1*, *AGP1*, *RPS5*, and *SCR1*, DNA fragments were amplified by PCR from yeast genomic DNA, purified, and ^32^P-labeled by random priming with the Klenow fragment (TOYOBO). The PCR primers used were as follows: *VTC1*, TK9030-TK9013; *CYC1*, TK9727-TK9745; *AGP1*, TK8195-TK8196; *RPS5*, TK493-TK494; and *SCR1*, TK9507-TK10081.

## Results

### A reporter system for analyzing Taf1p/TFIID- and/or Spt3p/SAGA- dependent CE function

We first sought to use the *CYC1* promoter to measure the activities of SAGA-dependent transcription from various CE sequences isolated by a random screen, in which this promoter was used as a backbone [51]. Consistent with a previous study [59], we confirmed that the *Δspt3* mutation significantly decreased *CYC1* transcription (S1 Fig). However, the effect of the *taf1* mutation was stronger than that of *Δspt3*: the former abolished the transient increase of *CYC1* transcription occurring at a later phase after temperature shift, whereas the latter failed to do so (S1 Fig). Therefore, it is likely that TFIID plays a more predominant and indispensable role in *CYC1* transcription than SAGA. If so, this promoter would not be adequate for the purpose of examining how efficiently SAGA mediates transcription from TATA-like element(s) in a manner independent of TFIID.

Fortuitously, we found that the *AGP1* promoter was more suitable for our purpose than the *CYC1* promoter because transcription of this gene, which encodes a low-affinity and broad-specificity amino acid permease [60, 61], decreased transiently after the temperature shift from 25°C to 37°C, and recovered in the *taf1* mutant strain to a greater extent than in the wild-type strain (S1 Fig). Importantly, *AGP1* transcription was abolished almost completely under the same conditions in the *taf1 Δspt3* double mutant strain, indicating that the transcriptional stimulation observed after the temperature shift in the *taf1* mutant strain must be supported by Spt3p/SAGA (S2 Fig). This situation is in stark contrast to *CYC1* transcription, which predominantly depends on Taf1p/TFIID, although it may be facilitated by Spt3p/SAGA (S1 and S2 Figs).

Two possible models could explain the function of TFIID and SAGA (S2C Fig). Namely, given that SAGA and TFIID bind to the UAS and core promoter, respectively [20, 21], SAGA may function upstream of (left panel) or in parallel to TFIID (right panel). Recent genome-wide studies [20, 21] support a sequential model (left panel), as transcription of nearly all class II genes depends on both TFIID and SAGA, whereas previous genome-wide studies [7, 34] supported a parallel model (right panel), as severe transcriptional defects were observed only when both factors were impaired. Currently, it remains unknown whether there are distinct types of promoters on which TFIID and SAGA function differently, as described here. Nevertheless, to determine how efficiently SAGA mediates transcription from TATA-like element(s) independently of TFIID, parallel-type promoters would be more adequate than sequential-type promoters because SAGA is more directly involved in transcription on the former than on the latter. The results described in S1 Fig suggest that the *CYC1* and *AGP1* promoters belong to the sequential and parallel types, respectively (S2A and S2B Fig). Therefore, we decided to measure Spt3p/SAGA-dependent transcriptional activities of various CE sequences using the *AGP1* promoter.

**Fig 1:**
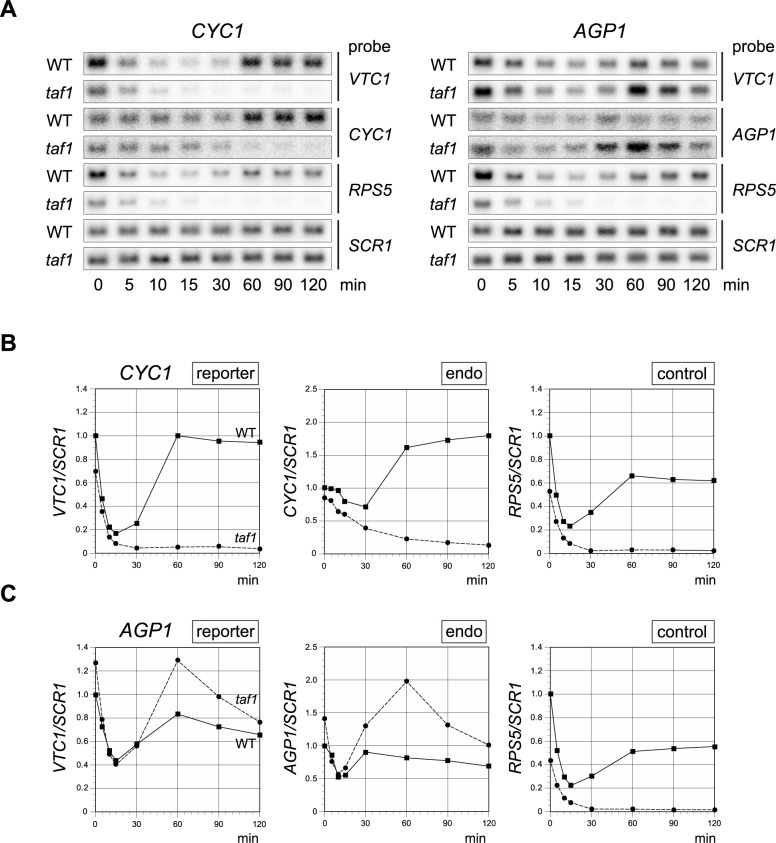
Effect of the *taf1* mutation on the expression kinetics of the *CYC1* and *AGP1* promoters, tested using the *VTC1* reporter system. **A**. Northern blot analyses to measure expression of the reporter (*VTC1*) or control genes (*CYC1*, *AGP1*, *RPS5*, and *SCR1*) in *TAF1* (hereafter, WT) or temperature-sensitive *taf1-N568Δ* (hereafter, *taf1*) strains carrying a reporter driven by the *CYC1* (left panel) or *AGP1* (right panel) promoter, as indicated above the blots. The strains used were YTK16396, YTK16397, YTK17952, and YTK17974 (S1 Table). These strains were grown to logarithmic phase at 25°C in YPD media (0 min) or further incubated at 37°C for the period of time indicated below the blots (5, 10, 15, 30, 60, 90, or 120 min). Gene-specific probes are indicated on the right. **B**. Raw data shown in the left panel of **A** were quantified and presented graphically. Values for each transcript (left: *VTC1*; center: *CYC1*; right: *RPS5*) from WT (closed squares connected by a solid line) or *taf1* (closed circles connected by a dashed line) strains were normalized against the level of *SCR1* (pol III transcript), and are presented relative to the corresponding values in the WT at 0 min (i.e., the *VTC1/SCR1*, *CYC1/SCR1*, and *RPS5/SCR1* ratios in the leftmost lane for the WT strain are defined as 1 in each graph). *RPS5* mRNA was used as a control to confirm that the temperature shift experiment was conducted appropriately. **C**. Raw data shown in the right panel of **A**, summarized as described in **B**.

To this end, we first tested whether Spt3p/SAGA-dependent transcription from the *AGP1* promoter could be recapitulated using the *VTC1* reporter system (Fig 1) [51, 62]. In these experiments, promoter activities were assessed by Northern blot analyses for *VTC1* mRNA, instead of by toluidine blue (TB) staining for accumulated polyphosphate in the vacuole, because Northern blotting enables more accurate measurement of transcription [25]. The results revealed that transcriptional recovery of the reporter gene driven by the *AGP1* promoter was stronger in the *taf1* strain than in the wild type after the temperature shift, consistent with the expression kinetics of the endogenous *AGP1* mRNA measured in the same cells (Fig 1A and 1C). We also confirmed that this transcriptional stimulation of the *AGP1* promoter after the temperature shift was dependent on Spt3p/SAGA in the *VTC1* reporter strain (S3 Fig). Furthermore, as expected based on our previous study [51], we confirmed that Taf1p/TFIID-dependent transcription from the *CYC1* promoter could also be recapitulated using this system, as the expression kinetics of the *VTC1* and endogenous *CYC1* mRNAs were almost similar to each other (Fig 1A and 1B). It should be noted that the transient decrease in the level of *VTC1* (reporter) mRNA after the temperature shift was greater than that of endogenous *CYC1* mRNA (Fig 1A and 1B). Although the cause of this difference is currently unknown, it may be related to differences in the stabilities of these two mRNAs. Collectively, these observations indicate that this system is suitable for analyzing CE function under conditions in which transcriptional activities depend predominantly on Taf1p/TFIID (*CYC1* promoter) or Spt3p/SAGA (*AGP1* promoter).

### Spt3p/SAGA mediates transcription from TATA-less promoters

**Fig 2:**
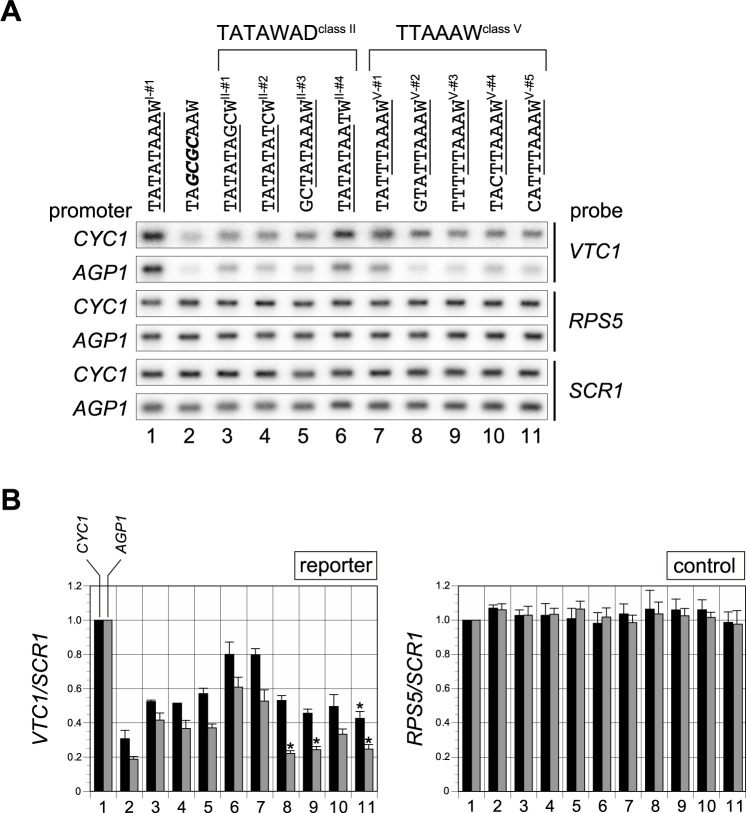
Transcriptional activities of several class II and class V core promoter element (CE) sequences in the *CYC1* and *AGP1* promoters. **A**. Northern blot analyses to examine expression of the reporter (*VTC1*) or control genes (*RPS5* and *SCR1*) in a WT strain carrying a reporter driven by the *CYC1* or *AGP1* promoter, as indicated on the left, in which the TATA box was replaced with one of several CE sequences of class II (TATAWAD, lanes 3–6) or V (TTAAAW, lanes 7–11), as indicated above the blots. TATATAAA (consensus TATA box, lane 1) or TA*GCGC*AA (lane 2) sequences were used as positive and negative controls, respectively. The portion of each sequence matching the consensus sequence of each class (i.e., TATAWAWR, TATAWAD, and TTAAAW) is underlined. “W” at the 3’-end of each CE sequence above the blot corresponds to “A” or “T” in the *CYC1* or *AGP1* promoter, respectively (i.e., the ninth base following the thickly underlined 8 bp sequence in S5 Fig). *CYC1* promoter-containing strains were YTK16396, 16398, 16408, 16410, 16412, 16414, 16400, 16402, 16404, 16406, and 16416. *AGP1* promoter-containing strains were YTK17952, 17954, 17964, 17966, 17968, 17970, 17956, 17958, 17960, 17962, and 17972 (S1 Table). These strains were grown to logarithmic phase at 25°C in YPD media. Gene-specific probes were indicated on the right. **B**. Raw data shown in **A** were quantified and presented graphically. Values for each transcript (left: *VTC1*; right: *RPS5*) from strains carrying *CYC1* (black bars) or *AGP1* (gray bars) promoter-driven reporters were normalized against the level of *SCR1* (pol III transcript) and are presented relative to the value for the TATA box (TATATAAAW^I-#1^, lane 1). Each bar represents the average of biological triplicates, with standard deviation. One representative experiment of three is shown in **A**. Asterisks in the left panel (lanes 8, 9, and 11) indicate statistically insignificant differences (p > 0.01, one-way ANOVA followed by post-hoc analysis with Tukey's honest significant difference test) relative to the value in the negative control (lane 2).

Previously, we showed that, in comparison with many other promoters in the yeast genome, the *CYC1* promoter more strongly prefers active CE sequences belonging to class II or class V [51]. Hence, we measured the transcriptional activities of several class II or V sequences in the *CYC1* or *AGP1* promoters (Fig 2). In the *CYC1* promoter, the activities of these sequences as measured by Northern blot analyses (Fig 2A) were well correlated with previous measurements made by TB staining (S4 Fig) [51]. Notably, all of the four class II sequences (II-#1, 2, 3, 4) and at least two class V sequences (V-#1, 4) exhibited significantly (p value < 0.01, one-way ANOVA followed by post-hoc analysis with Tukey's honest significant difference test) stronger CE activities than the negative control (TA*GCGC*AAW; lane 2) even in the *AGP1* promoter (Fig 2). Furthermore, these class II or V sequences had slightly weaker activities (calculated relative to the activity of the consensus TATA box; TATATAAAW^I-#1^) when they were tested in *AGP1* than in *CYC1* (compare black and gray bars in Fig 2B). These observations suggest that SAGA can mediate transcription not only from the TATA box but also from class II or V sequences, albeit less efficiently for the class V sequences. Alternatively, considering that both factors are involved in transcription from the *AGP1* promoter (S2 Fig), it remains possible that SAGA mediates transcription only from the TATA box, whereas TFIID does so from class II or V sequences.

**Fig 3:**
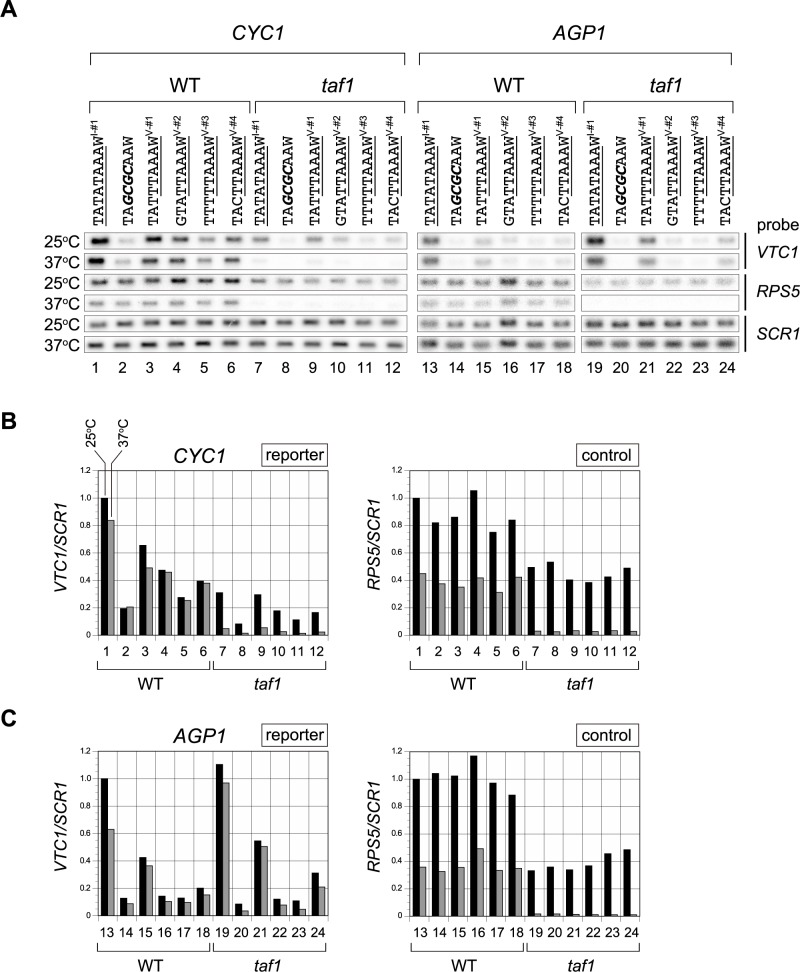
Taf1-dependence or -independence of transcription from several class V CE sequences in the *CYC1* and *AGP1* promoters. **A**. Northern blot analyses to examine expression of the reporter (*VTC1*) or control genes (*RPS5* and *SCR1*) in WT or *taf1* strains carrying a reporter driven by the *CYC1* or *AGP1* promoter in which the TATA box was replaced by one of several CE sequences of class V (TTAAAW, lanes 3–6, 9–12, 15–18, and 21–24), as indicated above the blots. TATATAAA (consensus TATA box: lanes 1, 7, 13, and 19) and TA*GCGC*AA (lanes 2, 8, 14, and 20) sequences were used as positive and negative controls, respectively. The portion of each sequence matching the consensus sequence of each class (i.e., TATAWAWR, TTAAAW) is underlined. “W” at the 3’-end of each CE sequence is as described in Fig 2A. *CYC1* promoter-containing strains were YTK16396, 16398, 16400, 16402, 16404, 16406, 1639, 16399, 16401, 16403, 16405, and 16407. *AGP1* promoter-containing strains were YTK17952, 17954, 17956, 17958, 17960, 17962, 17974, 17976, 17978, 17980, 17982, and 17984 (S1 Table). These strains were grown to logarithmic phase at 25°C in YPD media (indicated as “25°C” on the left), or further incubated at 37°C for 2 hours (lanes 1–12) or 1 hour (lanes 13–24) (indicated as “37°C” on the left). Gene-specific probes are indicated on the right. **B**. Raw data shown in the left panel of **A** were quantified and presented graphically. Values for each transcript (left: *VTC1*; right: *RPS5*) from WT or *taf1* strains cultured at 25°C (black bars) or 37°C (gray bars) were normalized against the level of *SCR1* (pol III transcript), and are presented relative to the value for the TATA box at 25°C (i.e., the *VTC1/SCR1* or *RPS5/SCR1* values for TATATAAAW^I-#1^ indicated to the left of lane 1). **C**. Raw data shown in the right panel of **A** are summarized as described in **B**.

To clarify this issue, we measured transcriptional activities of the four class V sequences (V-#1, 2, 3, 4) in the *CYC1* or *AGP1* promoters under the condition in which Taf1p/TFIID function was intact (WT) or impaired (*taf1*) (Fig 3). The results showed that functional impairment of Taf1p/TFIID decreased the CE activities of these four sequences in the *CYC1* promoter (Fig 3A and 3B) but not in the *AGP1* promoter (Fig 3A and 3C). As expected, *RPS5* transcription was greatly reduced in the *taf1* strain (control), confirming that Taf1p/TFIID function was impaired as expected under this experimental condition. Therefore, we conclude that Spt3p/SAGA can mediate transcription from the TATA-less promoters, at least those containing class V sequences such as TATTTAAAW^V-#1^ (lane 21 in Fig 3C) or TACTTAAAW^V-#4^ (lane 24 in Fig 3C), independently of Taf1p/TFIID.

### Taf1p/TFIID-dependence or -independence of transcription cannot be altered by variation of the TATATANN sequence

Previous studies showed that whether the creation of a TATA box could decrease the Taf1p-dependence of transcription from TATA-less promoters depended on promoter context, e.g., it could restore transcription of the *TUB2* promoter [58] but not that of the *RPS5* promoter [63, 64] in *taf1* strains. Moreover, another study reported that randomization of a very short (2 bp) flanking sequence of the TATA box greatly affected transcription from the *ENO2* and *PDC1* promoters [29], supporting the idea that TATA function depends on promoter context. Furthermore, the two TATA boxes in the *CYC1* promoter, i.e., TATAß [TATATATA] and TATAα [TATATAAA], were shown to be functionally different [65], even though this difference could not be recapitulated in the *VTC1* reporter system [51]. As shown in Figs 1–3 and S3 Fig, the TATATAAAW^I-#1^ sequence derived from the *CYC1* promoter was used as the TATA box even for the *AGP1* promoter whose original TATA box was TATATAAG (S5 Fig). These two TATA boxes could support Taf1p-independent transcription from the *AGP1* promoter (Figs 1 and 3; S1 and S3 Figs). However, given that Taf1p-dependence [58, 63, 64] or some as-yet-uncharacterized function [29, 65] of the TATA box depends on the promoter context or the sequence itself, as described above, it remains possible that some minor variation of the TATA sequence may influence the Taf1p-dependence or -independence of transcription from the *CYC1* and *AGP1* promoters.

**Fig 4:**
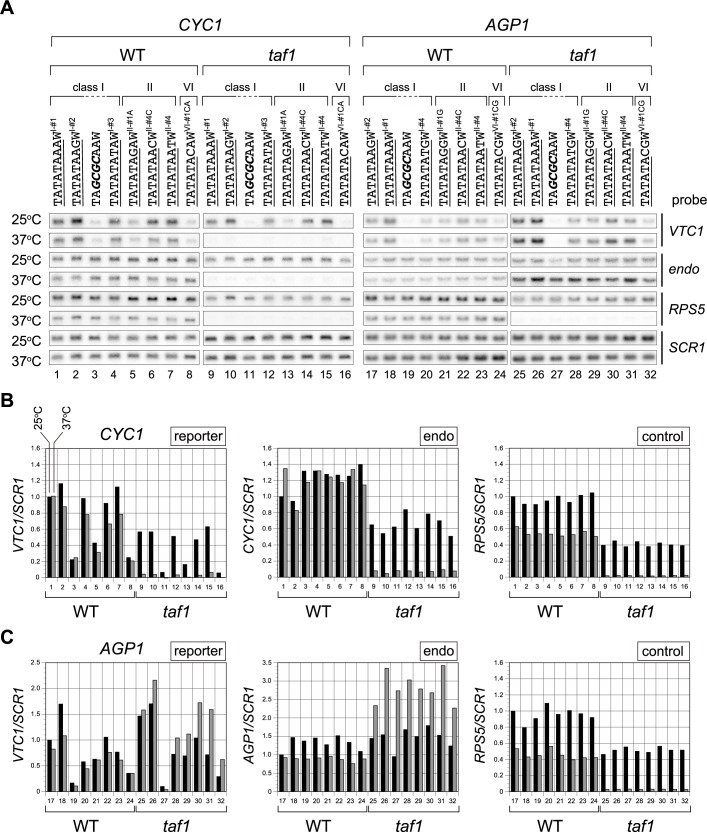
Taf1-dependence or -independence of transcription from several TATATANN sequences of classes I, II, and VI in the *CYC1* and *AGP1* promoters. **A**. Northern blot analyses to examine the expression of the reporter (*VTC1*) or control genes (*CYC1*, *AGP1*, *RPS5*, and *SCR1*) in WT or *taf1* strains carrying a reporter driven by the *CYC1* or *AGP1* promoter in which the TATA box was replaced with one of several TATATANN sequences of class I (TATAWAWR: lanes 1, 2, 4, 9, 10, 12, 17, 18, 20, 25, 26, and 28), II (TATAWAD: lanes 5–7, 13–15, 21–23, and 29–31), or VI (Wx6: lanes 8, 16, 24, and 32), as indicated above the blots. TATATAAA (*CYC1*-TATA; lanes 1, 9) / TATATAAG (*AGP1*-TATA; lanes 17, 25) and TA*GCGC*AA (lanes 3, 11, 19, 27) sequences were used as positive and negative controls, respectively. The portion of each sequence matching the consensus sequence of each class (i.e., TATAWAWR, TATAWAD, Wx6) is underlined. “W” at the 3’-end of each CE sequence is as described in Fig 2A. Identification tags for each sequence are indicated as a superscript according to CE class (i.e., I, II, or VI) and the dinucleotide sequence NN at the 3’-end of TATATANN to enable discernment of which sequences are identical or different among Figs 2, 3 and 4. *CYC1* promoter-containing strains were YTK16396, 17566, 16398, 17562, 17560, 17564, 17568, 17558, 16397, 17578, 16399, 17574, 17572, 17576, 17580, and 17570. *AGP1* promoter-containing strains were YTK17590, 17952, 17954, 17586, 17584, 17591, 17593, 17582, 17603, 17974, 17976, 17599, 17597, 17604, 17606, and 17595 (S1 Table). These strains were grown to logarithmic phase at 25°C in YPD media (indicated as “25°C” on the left) or further incubated at 37°C for 2 hours (lanes 1–16) or 1 hour (lanes 17–32) (indicated as “37°C” on the left). Gene-specific probes were indicated at the right. “*endo*” indicates *CYC1* (left panel) or *AGP1* (right panel) probes. **B**. Raw data shown in the left panel of **A** were quantified and presented graphically. Values for each transcript (left: *VTC1*; center: *CYC1*; right: *RPS5*) from WT or *taf1* strains cultured at 25°C (black bars) or 37°C (gray bars) were normalized against the level of *SCR1* (pol III transcript) and are presented relative to the value for the TATA box at 25°C (i.e., the *VTC1/SCR1*, *CYC1/SCR1*, or *RPS5/SCR1* ratios of TATATAAAW^I-#1^ indicated to the left of lane 1). **C**. Raw data shown in the right panel of **A** are summarized as described in **B**, except that the values are presented relative to the value for TATATAAGW^I-#2^ at 25°C indicated to the left of lane 17.

To explore this possibility, we measured transcriptional activities of seven TATATANN sequences in each promoter in cells in which Taf1p/TFIID function was intact (WT) or impaired (*taf1*) (Fig 4). The TATATANN sequence was chosen for these studies because the difference between TATAß [TATATATA] and TATAα [TATATAAA] is located within the two 3’-terminal bases “NN”. Specifically, the same set of four TATATANN sequences (NN: AG [I-#2], AA [I-#1], AC [II-#4C], AT [II-#4]) were tested in both promoters, whereas the remaining three differed slightly between promoters, as follows: NN = TA [I-#3], GA [II-#1A], or CA [VI-#1CA] for the *CYC1* promoter; and NN = TG [I-#4], GG [II-#1G], or CG [VI-#1CG] for the *AGP1* promoter. Thus, the most 3’-terminal nucleotide (A or G) was replaced by the original one (NN: AA in *CYC1* and AG in *AGP1*) (S5 Fig). Note that the identification tags in square brackets were assigned according to CE class [i.e., I (TATAWAWR), II (TATAWAD), or VI (Wx6)], as well as to the content of the dinucleotide sequence (NN), to enable discernment of which sequences were identical or different in Figs 2, 3 and 4.

The results, categorized by class (I, II, and VI) (Fig 4), showed that transcription from all active CE sequences in the *CYC1* promoter was Taf1p-dependent, while that from those in the *AGP1* promoter was Taf1p-independent. Namely, no TATATANN sequences, as far as we examined here, could alter the Taf1p-dependence or -independence of transcription from these two promoters. Therefore, we conclude that Spt3p/SAGA can mediate transcription from the *AGP1* promoter carrying CE sequences belonging not only to class V (Fig 3) but also to class II or VI (Fig 4) independently of Taf1p/TFIID. Notably, in the *AGP1* promoter the TATATACGW^VI-#1CG^ sequence (lanes 24 and 32) exhibited weak activity that was nonetheless significantly stronger than that of the negative control (TA*GCGC*AAW) (lanes 19 and 27). TATATAC-containing sequences were rarely isolated in the original screen [51], as inferred from the class II consensus sequence (i.e., TATAWAD). Consistent with this, in the *CYC1* promoter the TATATACAW^VI-#1CA^ sequence (lanes 8 and 16) did not exhibit stronger activity than the negative control (lanes 3 and 11). These observations suggest that TATATAC might be a Spt3p/SAGA-specific CE motif.

### Taf1p/TFIID-dependence or -independence is conferred by the UAS

**Fig 5:**
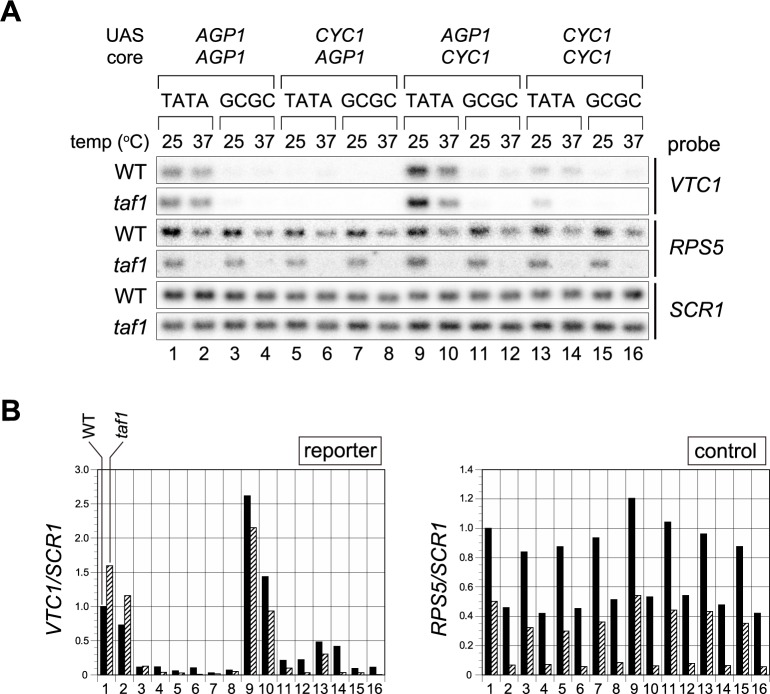
Taf1p-dependence or -independence of transcription is conferred by the upstream activating sequence (UAS) of the *CYC1* or *AGP1* promoters. **A**. Northern blot analyses to examine the expression of the reporter (*VTC1*) or control genes (*RPS5* and *SCR1*) in WT or *taf1* strains carrying a reporter driven by the *CYC1* promoter (i.e., *CYC1*_*UAS*_ [-400 –-231 bp] + *CYC1*_*core*_ [-230 –+1 bp], lanes 13–16), the *AGP1* promoter (i.e., *AGP1*_*UAS*_ [-780 –-301 bp] + *AGP1*_*core*_ [-300 –+1 bp], lanes 1–4), or chimeric promoters such as *CYC1*_*UAS*_+*AGP1*_*core*_ (lanes 5–8) or *AGP1*_*UAS*_+*CYC1*_*core*_ (lanes 9–12), as indicated above the blots. Nucleotide coordinates are relative to the A (+1) of the initiation codon ATG. All four promoters contain TATATAAA (lanes 1–2, 5–6, 9–10, 13–14) or TA*GCGC*AA (lanes 3–4, 7–8, 11–12, 15–16) sequences at the TATA site, as indicated below the index for “UAS” and “core”. *TAF1* strains were YTK17952, 17954, 17766, 17769, 17748, 17750, 16396, and 16398; *taf1* strains were YTK17974, 17976, 17802, 17804, 17784, 17786, 16397, and 16399 (S1 Table). These strains were grown to logarithmic phase at 25°C in YPD media (indicated as “25” above the blot) or further incubated at 37°C for 2 hours (indicated as “37” above the blot). Gene-specific probes are indicated at the right. **B**. Raw data shown in **A** were quantified and presented graphically. Values for each transcript (left: *VTC1*; right: *RPS5*) derived from WT (black bars) or *taf1* (gray bars) strains were normalized against the level of *SCR1* (pol III transcript), and are presented relative to the value for the TATA box of the *AGP1* promoter at 25°C (i.e., the *VTC1/SCR1* or *RPS5/SCR1* values indicated to the left of lane 1).

Previous work showed that the Taf1p-dependence of transcription could be conferred by the core promoter [64, 66], the UAS [67], or both [22, 63], depending on the promoter context. The results described above indicated that Taf1p-dependence or -independence is highly robust with respect to variation of the TATA sequence, at least in the *CYC1* or *AGP1* promoters (Fig 4). Hence, we next sought to determine which region, e.g., the core promoter or UAS, could confer Taf1p-dependence or -independence on these two promoters. For this purpose, we constructed two additional chimeric promoters, i.e., *AGP1*_*UAS*_*+CYC1*_*core*_ and *CYC1*_*UAS*_*+AGP1*_*core*_, by connecting *AGP1*_*UAS*_ [-780 –-301 bp] or *CYC1*_*UAS*_ [-400 –-231 bp] to *CYC1*_*core*_ [-230 –+1 bp] or *AGP1*_*core*_ [-300 –+1 bp], respectively [68, 69]. Transcriptional activities of these two chimeric promoters, as well as those of the original *CYC1* and *AGP1* promoters, were measured in cells in which Taf1p/TFIID function was intact (WT) or impaired (*taf1*) (Fig 5). The results clearly showed that the *AGP1*_*UAS*_*+CYC1*_*core*_ promoter was more active than the *AGP1* promoter, and that both promoters were expressed in a Taf1p-independent manner (lanes 1–2 and 9–10). However, it was unclear whether the *CYC1*_*UAS*_*+AGP1*_*core*_ promoter was expressed in a Taf1p-dependent manner (lanes 5–6), similar to the *CYC1* promoter (lanes 13–14), because the activity of the *CYC1*_*UAS*_*+AGP1*_*core*_ promoter was weaker than that of the *CYC1* promoter under these experimental conditions.

To clarify this point, we measured the activities of these promoters in media containing raffinose instead of glucose. As expected, transcription of the two promoters containing *CYC1*_*UAS*_ was stimulated by raffinose, presumably due to release from glucose repression (compare lanes 1–4 and 9–12 in S6A and S6B Fig) [70]. Next, the activity of the *CYC1*_*UAS*_*+AGP1*_*core*_ promoter was measured again in cells in which Taf1p/TFIID function was intact (WT) or impaired (*taf1*) (S6C and S6D Fig). Unexpectedly, transcription from this promoter was not stimulated by raffinose in the *taf1* strain, even at 25°C (lanes 5 and 13 in S6C and S6D Fig). However, transcription from the *CYC1* promoter could be stimulated by raffinose in the *taf1* strain at 25°C, but not at 37°C (data not shown). These observations suggest that activation of either *AGP1*_*core*_ or *CYC1*_*core*_ by *CYC1*_*UAS*_ is Taf1p-dependent, but the *AGP1*_*core*_ requires more integral Taf1p function than the *CYC1*_*core*_. More importantly, the results obtained after optimizing the experimental conditions (e.g., using fresh probe and/or exposing for a longer time) clearly showed that the *CYC1*_*UAS*_*+AGP1*_*core*_ promoter could be expressed in a Taf1p-dependent manner (lanes 5–6 in S6C and S6D Fig), similar to the *CYC1* promoter (lanes 13–14 in Fig 5). Based on these findings, we conclude that the Taf1p-dependence or -independence of these two promoters was conferred by *CYC1*_*UAS*_ or *AGP1*_*UAS*_, respectively.

### Core promoter sequences other than the TATA box may determine the function of Spt3p/SAGA on the *AGP1* promoter after inactivation of Taf1p/TFIID

**Fig 6:**
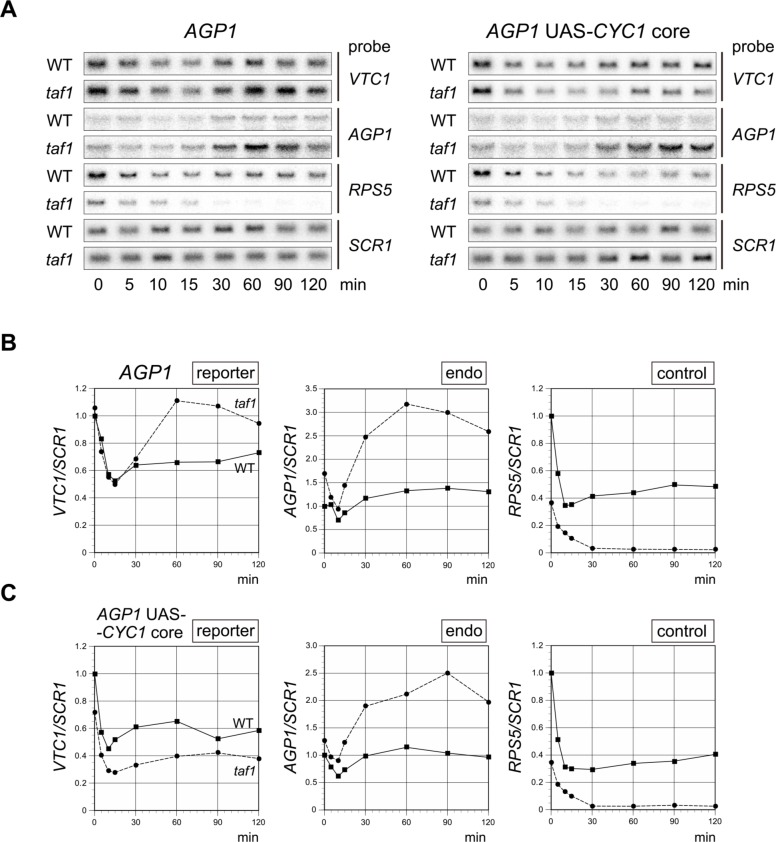
Spt3p-dependent transcriptional stimulation after the temperature shift in the *taf1* strain occurs specifically on the *AGP1* core promoter. **A**. Northern blot analyses to examine the expression of the reporter (*VTC1*) or control genes (*AGP1*, *RPS5*, and *SCR1*) in WT or *taf1* strains carrying a reporter driven by the *AGP1* (left panel) or chimeric *AGP1*_*UAS*_+*CYC1*_*core*_ (right panel) promoter. Experiments were conducted as described in Fig 1A. The strains used were YTK17952 and 17974 (left panel) and YTK17748 and 17784 (right panel). **B**. Raw data shown in the left panel of **A** were quantified and presented graphically as described in Fig 1B. Note that transcriptional stimulation in the *taf1* strain after the temperature shift was highly reproducible, i.e., the results were consistent with those shown in Fig 1C. **C**. Raw data shown in the right panel of **A** were quantified and presented graphically as described in Fig 1B. Note that transcriptional stimulation of the *CYC1* core promoter after the temperature shift was not observed in the *taf1* strain.

As described above, transcription from the *AGP1* promoter was stimulated to a greater extent in the *taf1* strain than in the wild type after the temperature shift (Fig 1). In addition, the chimeric *AGP1*_*UAS*_*+CYC1*_*core*_ promoter, which is more active than the *AGP1* promoter, was also expressed in a Taf1p-independent manner (Fig 5). Hence, we next investigated whether transcription from the *AGP1*_*UAS*_*+CYC1*_*core*_ promoter exhibited similar kinetics to that of the *AGP1* promoter after the temperature shift (Fig 6). Surprisingly, the results revealed that the expression kinetics of these two promoters were quite different. Namely, stronger *taf1* strain-specific transcriptional stimulation after the temperature shift was reproducibly observed for the *AGP1* promoter (Fig 6A and 6B), but not for the *AGP1*_*UAS*_*+CYC1*_*core*_ promoter (Fig 6A and 6C). Transcription from the *AGP1*_*UAS*_*+CYC1*_*core*_ promoter was instead weakened by the *taf1* mutation under the same conditions (Fig 6A and 6C).

**Fig 7:**
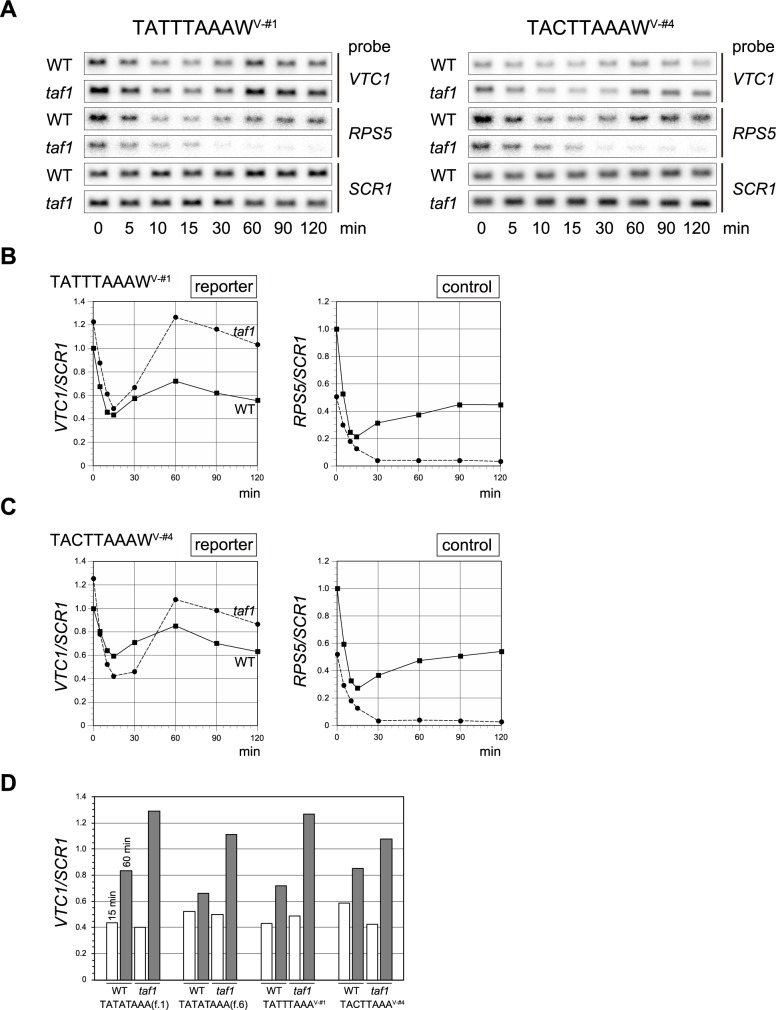
Spt3p-dependent transcriptional stimulation after the temperature shift in the *taf1* strain occurs on *AGP1* core promoters whose TATA boxes were replaced with class V CE sequences. **A**. Northern blot analyses to examine the expression of the reporter (*VTC1*) or control genes (*RPS5* and *SCR1*) in WT or *taf1* strains carrying a reporter driven by the *AGP1* promoter in which the TATA box was replaced with TATTTAAA (left panel) or TACTTAAA (right panel) sequences. Experiments were conducted as described in Fig 1A. The strains used were YTK17956 and 17978 (left panel), and YTK17962 and 17984 (right panel). **B**. Raw data shown in the left panel of **A** were quantified and presented graphically as described in Fig 1B, except that measurement of an endogenous gene was omitted. **C**. Raw data shown in the right panel of **A** were quantified and presented graphically as described in Fig 1B, except that the measurement of an endogenous gene was omitted. **D**. Normalized *VTC1/SCR1* ratios at 15 min (open bars) or 60 min (gray bars) after the temperature shift, adapted from the plots in Figs 1C (first four lanes), 6B (second four lanes), 7B (third four lanes) and 7C (fourth four lanes), are summarized in this figure to confirm that Spt3p-dependent transcriptional stimulation occurs on the *AGP1* promoter regardless of whether it contains a TATA box (TATATAAA) or a TATA-like element (TATTTAAA or TACTTAAA). In each set, the values are presented relative to those of the WT at 0 min (defined as 1). Note that the ratios of the value at 60 min to the value at 15 min are always higher in the *taf1* strain than in the WT, indicating that Spt3p-dependent transcriptional stimulation after the temperature shift occurred on all promoters tested here.

Fig 3 shows that SAGA could mediate transcription from two class V CE sequences, i.e., TATTTAAAW^V-#1^ and TACTTAAAW^V-#4^. To determine whether the TATA box is required for Spt3p/SAGA-dependent transcriptional stimulation in the *taf1* strain after the temperature shift, we examined the expression kinetics of transcription from these two CEs, as described above for the *AGP1* and *AGP1*_*UAS*_*+CYC1*_*core*_ promoters (Fig 7A–7C). The results revealed a significant *taf1* strain-specific transcriptional stimulation for both CEs, particularly when the relative ratios of the value obtained at 60 min to that at 15 min were compared between WT and *taf1* strains (Fig 7D). Therefore, we conclude that, although Taf1p/TFIID-independence of *AGP1* transcription is conferred by *AGP1*_*UAS*_, Spt3p/SAGA-dependent transcriptional stimulation of this promoter after inactivation of Taf1p/TFIID is directed by *AGP1*_*core*_ sequences other than the TATA element.

## Discussion

Previously, we showed that the chimeric *UAS*_*GAL*_*+CYC1* promoter on a plasmid could be activated by various types of activation domains (e.g., those derived from Abf1p, Gal4p, Gcn4p, Adr1p, Rap1p, TAND1, EBNA2, or VP16) in a Taf1p-independent manner, unless the TATA sequence was mutated [58, 63]. Consistent with this, other groups also reported that the *CYC1* promoter belongs to a SAGA-dependent and TFIID-independent class [59, 66]. However, the criteria used in those studies were not especially stringent: one group tested the Taf1p-dependence of transcription only under mild conditions (e.g., 30°C in the *taf1* strain) [66], whereas the other group did not test the Taf1p-dependence of transcription itself, but instead examined Taf1p occupancy on this promoter [59]. On the other hand, genome-wide expression analyses demonstrated that the *CYC1* promoter is TFIID-dominated and not SAGA-dominated [7]. In a more recent study, we showed that the *CYC1* promoter was Taf1p-dependent at the *VTC1* reporter locus, but not at the endogenous *CYC1* locus [51]. During the course of the current study, however, we noticed that the data for *CYC1* mRNA derived from the *CYC1* locus obtained at that time, cited as “data not shown” in the original reference [51], were contaminated with the signal from *SCR1* RNA due to insufficient washing of the *Northern* blot prior to reprobing. After repeating the same experiment several times, we are now convinced that the *CYC1* promoter is Taf1p-dependent even at the endogenous *CYC1* locus (S1 Fig), whereas it is Taf1p-independent in the context of the chimeric *UAS*_*GAL*_*+CYC1* promoter on a plasmid. Although it remains unclear why only the chimeric *UAS*_*GAL*_*+CYC1* promoter exhibited Taf1p-independence, it is possible that Taf1p/TFIID function is specifically required for activity of the *CYC1* promoter in the chromatin environment. In fact, recent studies suggest that the +1 nucleosome or its repositioning may differentially affect the two PIC assembly pathways mediated by TFIID and SAGA [8, 71, 72]. Consistent with this, TBP binding to the *CYC1* promoter is stimulated by activators when it is on a plasmid [73] but not when it is integrated into the genome [74], suggesting that TBP preloading and pol II release from the poised state upon activation, which are characteristic features of this promoter [59, 75], depend on the chromatin environment and Taf1p/TFIID function.

### SAGA mediates transcription from the TATA-like element independently of TFIID

In this study, we set up a *VTC1* reporter system using the *AGP1* promoter to test for Spt3p/SAGA-dependent transcription from various types of CE sequences without the involvement of Taf1p/TFIID. In this system, following the temperature shift to inactivate the mutated version of Taf1p/TFIID, transcription transiently decreased and then recovered to a much greater extent in the *taf1* strain than in the WT in a Spt3p/SAGA-dependent manner (Fig 1, S3 Fig). Because an increase in mRNA level strongly implies *de novo* mRNA synthesis, the accumulation of larger amounts of mRNA in the *taf1* strain could be regarded as a *bona fide* product of Spt3p/SAGA-mediated transcription. Thus, even though the amount of newly synthesized mRNA [18, 46–49, 72, 76] was not measured, this system minimizes the possibility that mRNA remained due to elevated stability without any increase in transcription. Using this novel system, we found that several CE sequences belonging not only to class I (consensus TATA box; TATATAAAW^I-#1^, TATATAAGW^I-#2^, TATATATGW^I-#4^) but also to class II (TATAWAD; TATATAGGW^II-#1G^, TATATAACW^II-#4C^, TATATAATW^II-#4^), class V (TTAAAW; TATTTAAAW^V-#1^, TACTTAAAW^V-#4^), or class VI (Wx6; TATATACGW^VI-#1CG^) (the region of each sequence matching the consensus in each class is underlined) were transcriptionally active in the *AGP1* promoter even after inactivation of Taf1p/TFIID. These observations provide strong evidence that Spt3p/SAGA mediates transcription from the TATA-like element independently of Taf1p/TFIID.

Very recent genome-wide studies showed that TFIID and SAGA bind to the core promoter region and UAS, respectively, and mediate transcription from the promoters of nearly all class II genes [19–21]. In these studies, factor binding was monitored by ChEC-seq (chromatin endogenous cleavage coupled with high-throughput sequencing) [77], whereas transcriptional activity was assessed by measuring the amount of newly synthesized mRNA. By contrast, the longstanding and widely accepted view that TFIID and SAGA function predominantly in transcription from TATA-less and TATA box-containing promoters, respectively, was established based on measurements of steady-state mRNA levels [7]. As noted above, a buffering effect maintains global cytoplasmic mRNA levels upon transcriptional impairment [18, 20, 21, 46–49]. Thus, transcriptional defects of TATA-less or TATA box-containing promoters are likely to be restored unevenly by such an effect in TFIID or SAGA-defective strains. More specifically, because the defects of TATA-less promoters may be restored more completely in a SAGA-defective strain than in a TFIID-defective strain, the defects would be observable specifically in the TFIID-defective strain, whereas the defects of TATA box-containing promoters may be restored in the opposite fashion. If so, the mechanisms by which TFIID and SAGA mediate transcription differ for these two types of promoters, at least from the standpoint of “restorability” by such a buffering effect. Consistent with this, SAGA yielded stronger ChEC-seq signals at more upstream regions relative to the initiation site on TATA box-containing promoters than on TATA-less promoters [21]. Furthermore, depletion of Taf4p/TFIID from the nucleus decreased the amounts of newly synthesized mRNA to a greater extent from TATA-less promoters than from TATA box-containing promoters [20]. Therefore, as a next step, it will be crucial to more precisely determine the mechanisms by which TFIID and SAGA mediate transcription from these two types of promoters. The *CYC1* and *AGP1* promoters would provide useful model systems for this purpose because TFIID and SAGA function very differently on these two promoters, even if they both originally contained TATA boxes (S2 Fig).

### SAGA mediates transcription in a manner dependent on a core promoter structure other than the TATA box

Our mapping experiments using chimeric promoters revealed that the Taf1p-dependence and -independence of transcription from the *CYC1* and *AGP1* promoters, respectively, was conferred by the UAS rather than the core promoter (Fig 5, S6 Fig). Similarly, Taf1p-dependence was previously mapped to the UAS by comparing the *LexA-RPS5*_*core*_ (Taf1p-independent) and *RPS5*_*UAS*_*-RPS5*_*core*_ (Taf1p-dependent) promoters [67]. By contrast, Taf1p-dependence was mapped to the core promoter based on comparison between *RPS5*_*UAS*_*-ADH1*_*core*_ (Taf1p-independent) and *RPS5*_*UAS*_*-RPS5*_*core*_ (Taf1p-dependent) promoters [64, 67]. Comparison of the four chimeric promoters *2xGAL4 sites-GAL1*_*core*_ (Taf1p-independent), *RPS5*_*UAS*_*-GAL1*_*core*_ (Taf1p-independent), *2xGAL4 sites-RPS5*_*core*_ (Taf1p-dependent), and *RPS5*_*UAS*_*-RPS5*_*core*_ (Taf1p-dependent) also supported the latter conclusion that the core promoter is responsible for Taf1p-dependence [66]. Alternatively, Taf1p-dependence could be also conferred by the combination of the UAS and core promoter [22, 63].

In these studies, Taf1p-dependence of activation by *RPS5*_*UAS*_ was observed only for the TATA-less *RPS5*_*core*_ [64, 66, 67], but not for the TATA-containing *ADH1*_*core*_ [64, 67], *GAL1*_*core*_ [66], or *CYC1*_core_ [63]. A recent study showed that transcriptional repression of ribosomal protein genes (RPGs) under stressed conditions (including temperature shift, often used for inactivation of temperature-sensitive Taf proteins) occurs through the eviction of PIC and the Hmo1p-Ifh1p-Sfp1p complex, followed by repositioning of the +1 nucleosome to the upstream PIC assembly site [78, 79]. Because *RPG* transcription usually recovers within 1 hour after the temperature shift (Fig 1), Taf1p-dependence of *RPS5*_*core*_ activation by *RPS5*_*UAS*_ is likely to result from competitive binding of TFIID to overcome the repressive effect by repositioning the +1 nucleosome. Given that Taf1p was also recruited on *ADH1*_*core*_ by *RPS5*_*UAS*_ [67], Taf1p-independent *ADH1*_*core*_/*GAL1*_*core*_/*CYC1*_core_ activation by *RPS5*_*UAS*_ could be explained by a model in which, once TFIID is evicted by *RPS5*_*UAS*_ under temperature stress condition, TBP alone delivered by SAGA binds to these TATA-containing core promoters, probably because the TATA sites are vacant due to inappropriate repositioning of the +1 nucleosome. Consistent with this model, *ADH1*_*core*_ activation by *RPS5*_*UAS*_ is dependent on Taf6p, a common subunit of TFIID and SAGA [67]. Similar to *RPS5* transcription, a transient decrease in activity and Taf1p-dependent recovery after the temperature shift was also observed for the *CYC1* promoter (Fig 1). However, in the case of this promoter, Taf1p-dependence was mapped to *CYC1*_*UAS*_ instead of *CYC1*_*core*_. Furthermore, *CYC1*_*core*_ and *AGP1*_*core*_ both contain TATA boxes, and minor variation in the TATA sequence did not affect the Taf1p-dependence or -independence of these two promoters (Figs 3 and 4). Therefore, the mechanisms responsible for conferring Taf1p-dependence on the *RPS5* and *CYC1* promoters must be quite different.

Intriguingly, Spt3p/SAGA-dependent transcriptional stimulation of the *AGP1* promoter in the *taf1* strain after the temperature shift was specific to *AGP1*_*core*_ (Fig 6). Furthermore, this transcriptional stimulation was not compromised by the substitution of the TATA box with either of the two TATA-like elements (Fig 7), indicating that a region other than the TATA box is a critical determinant for the function of Spt3p/SAGA on this promoter. Two possible models could explain these observations. SAGA could specifically recognize *AGP1*_*core*_ to stimulate transcription after inactivation of TFIID. Alternatively, SAGA itself could be non-selective for *CYC1*_*core*_ or *AGP1*_*core*_, but inactivated Taf1p/TFIID could somehow inhibit SAGA specifically on *CYC1*_*core*_ so that SAGA cannot stimulate transcription from that sequence. Unfortunately in this regard, Taf1p occupancy was very low at the *CYC1* promoter [8, 59]. In addition, SAGA is usually recruited to the UAS, and its occupancy at the core promoter is difficult to detect, probably due to its highly dynamic nature [6, 18, 20, 21, 39, 80, 81]. Therefore, it may be difficult to distinguish between these two possibilities.

A recent study demonstrated that the difference in “regulatability” (regulatory amplitude conferred by the activator) [82] between TFIID-dominated and SAGA-dominated genes was due to the core promoter type rather than the number or type of activators [72]. Notably, the TATA box is not the sole determinant of core promoter type in regard to regulatability [72]. Consistent with this, another recent study using an *in vitro* transcription system suggested that some other features, in addition to the TATA box, may determine the difference in the ability of TBP to substitute for TFIID on TATA box-containing vs. TATA-less promoters [28]. In addition, the core promoters of TFIID- and SAGA-dominated genes have different free-energy landscapes [83, 84]. Considering that TFIID and SAGA bind to the promoters of nearly all class II genes [20, 21], it is likely that the function of these two factors is regulated by the core promoter structure at certain post-recruitment step(s).

In summary, we showed here that *AGP1*_*UAS*_ activated *CYC1*_*core*_ and *AGP1*_*core*_ in a Taf1p/TFIID-independent manner (Fig 5, S6 Fig) via different mechanisms (Fig 6). Similarly, *CYC1*_*UAS*_ activated *CYC1*_*core*_ and *AGP1*_*core*_ in a Taf1p/TFIID-dependent manner (Fig 5, S6 Fig), also via different mechanisms, with the *AGP1*_*core*_ activation requiring more integral function of Taf1p/TFIID than the *CYC1*_*core*_ activation (data not shown). Therefore, we propose that the functions of TFIID and SAGA are regulated in two steps: first, the UAS specifies TFIID or SAGA as the predominant factor on a given promoter, and then the core promoter structure guides this factor to conduct transcription in an appropriate manner. Future studies should seek to reveal the detailed mechanisms by which TFIID and SAGA mediate transcription from TATA box-containing and TATA-less promoters, as well as the physiological significance of the promoter-specific functions of these two related transcription complexes.

## Supporting information

S1 Fig Effect of the *taf1* and/or *Δspt3* mutation on the expression kinetics of the endogenous *CYC1* and *AGP1* promoters. **A**. Northern blot analyses to examine expression of *CYC1*, *AGP1*, or control genes (*RPS5* and *SCR1*) in four strains: *TAF1 SPT3* (indicated as WT in **B**; YTK11705), *taf1-N568Δ SPT3* (indicated as *taf1* in **B**; YTK11708), *TAF1 Δspt3* (indicated as *Δspt3* in **B**; YTK11871), or *taf1-N568Δ Δspt3* (indicated as *taf1 Δspt3* in **B**; YTK11873). The analyses were conducted as described in Fig 1A. **B**. Raw data shown in **A** were quantified and presented graphically as described in Fig 1B.
(EPS)

S2 Fig Model of the roles of TFIID and SAGA on the *CYC1* and *AGP1* promoters. **A**. Model of the roles of TFIID and SAGA on the *CYC1* promoter. Transcription from the *CYC1* promoter was significantly weakened in the *taf1* strain after the temperature shift (II), whereas it was also weakened in the *Δspt3* strain, but in a temperature shift-independent manner (III) (S1 Fig). Notably, transcription increased slightly at a later phase after the temperature shift in the *Δspt3* strain. Furthermore, transcription from this promoter was almost abolished in the *taf1 Δspt3* strain (IV) (S1 Fig), indicating that TFIID plays a predominant and more indispensable role in *CYC1* transcription, whereas SAGA plays a supportive and less indispensable role, i.e., it assists TFIID or other factors in transcription from this promoter in a WT strain (I). **B**. Model of the roles of TFIID and SAGA on the *AGP1* promoter. Transcription from the *AGP1* promoter was stimulated after the temperature shift in the *taf1* strain (II) but not in the *Δspt3* strain (III), whereas it was almost abolished in the *taf1 Δspt3* strain (IV) (S1 Fig). These observations indicate that TFIID and SAGA play redundant and/or antagonistic roles in *AGP1* transcription in the WT strain (I). Importantly, it is possible to determine whether Spt3p/SAGA mediates transcription from various CE sequences by assaying their transcriptional activities under condition II. **C**. Two possible models of the function of TFIID and SAGA. SAGA may function upstream of TFIID (left panel) or in parallel to TFIID (right panel). Parallel-type promoters (right panel) were more suitable for our purpose than sequential-type promoters (left panel) because SAGA is more directly involved in transcription on the former than on the latter.
(EPS)

S3 Fig Effect of a *taf1* and/or *Δspt3* mutation on the expression kinetics of the *AGP1* promoter when tested using the *VTC1* reporter system. **A**. Northern blot analyses to examine the expression of the reporter (*VTC1*) or control genes (*AGP1*, *RPS5*, and *SCR1*) in four strains: *TAF1 SPT3* (indicated as WT in **B**; YTK18974), *taf1-N568Δ SPT3* (indicated as *taf1* in **B**; YTK18986), *TAF1 Δspt3* (indicated as *Δspt3* in **B**; YTK18998), or *taf1-N568Δ Δspt3* (indicated as *taf1 Δspt3* in **B**; YTK19010). Analyses were conducted as described in Fig 1A. **B**. Raw data shown in **A** were quantified and presented graphically as described in Fig 1B.
(EPS)

S4 Fig Transcriptional profiles of several class II or V sequences tested in this study, measured previously by toluidine blue (TB) staining. **A**. Four CE sequences (marked with asterisks at the left) belonging to class II (TATATAGCA [#1], TATATATCA [#2], GCTATAAAA [#3], and TATATAATA [#4] isolated by a previous screen [51]) were tested in Figs 2 or 4 as TATATAGCW^II-#1^, TATATATCW^II-#2^, GCTATAAAW^II-#3^, and TATATAATW^II-#4^, respectively. The portion of each sequence matching the consensus TATAWAD is underlined in the preceding sentence or indicated by a red rectangle in the figure. This figure is adapted from S2 Fig of the original study [51]. The color code (left panel) and normalized isolation frequency (right panel) are as described previously [51]. **B**. Five CE sequences (marked with asterisks at the left) belonging to class V (TATTTAAAA [#1], GTATTAAAA [#2], TTTTTAAAA [#3], TACTTAAAA [#4], and CATTTAAAA [#5]) isolated in a previous screen [51] were tested in Figs 2 or 3 as TATTTAAAW^V-#1^, GTATTAAAW^V-#2^, TTTTTAAAW^V-#3^, TACTTAAAW^V-#4^, and CATTTAAAW^V-#5^, respectively. The portion of each sequence matching the consensus TTAAAW is underlined in the preceding sentence or indicated by a red rectangle in the figure. This figure is adapted from S3 Fig of the original study [51]. The color code (left panel) and normalized isolation frequency (right panel) are as described previously [51].
(EPS)

S5 Fig Nucleotide sequences of the *CYC1* and *AGP1* promoters. **A**. Nucleotide sequence of the *CYC1* promoter. The positions of the two TATA boxes and two TATA-like elements are underlined: #1 (TATA_β_), #2 (TATA_α_), #3, and #4. Each TATA box or TATA-like element was replaced with a specific sequence, denoted as “m”, to disrupt its transcriptional activity [51, 65]. The substituted nucleotides that differ from those in the WT are shown in bold italic font. The initiation codon ATG is marked with an open square along with the number +1 (indicating A of ATG). The arrows above the sequence indicate the transcriptional start site(s) (TSSs), which depend on TATA_β_ located at site #2 (marked with a thick underline). In this study, TATA_β_ was replaced with various CE sequences belonging to class I, II, V, or VI. **B**. Nucleotide sequence of the *AGP1* promoter. The position of the TATA box in the *AGP1* promoter is thickly underlined. This element was modified as described in **A**. Note that it was also replaced with TATA_β_ (TATATAAA) of the *CYC1* promoter and used as a positive control in all experiments, except in Fig 4, in which TATATAAG (original TATA box) was also tested in parallel. TSS and ATG are marked as described in **A**.
(EPS)

S6 Fig Taf1p-dependence of transcription from the *CYC1* promoter is conferred by the upstream activating sequence (UAS). **A**. Northern blot analyses to examine the expression of the reporter (*VTC1*) or control genes (*RPS5* and *SCR1*) in the WT strain carrying the reporter driven by the *CYC1* (lanes 1, 2, 9, and 10), *AGP1* (lanes 7, 8, 15, and 16), *CYC1*_*UAS*_+*AGP1*_*core*_ (lanes 3, 4, 11, and 12), or *AGP1*_*UAS*_+*CYC1*_*core*_ (lanes 5, 6, 13, and 14) promoters, as indicated at the top. All four promoters contain TATATAAA (odd numbered lanes) or TA*GCGC*AA (even-numbered lanes) sequences at the TATA site, as indicated below the index for “UAS” and “core”. Total RNA (20 μg) was isolated from these eight strains (i.e., YTK16396/16398/17766/17769/17748/17750/17952/17954) grown to a logarithmic phase at 25°C (lanes 1–8) or 30°C (lanes 9–16) in rich media containing 2% glucose (lanes 1–8) or 4% raffinose (lanes 9–16), blotted onto the membrane, and hybridized with the gene-specific probes indicated at the right. **B**. Raw data shown in **A** were quantified and presented graphically. Values for each transcript (*VTC1*/left graph, *RPS5*/right graph) were normalized against the level of *SCR1* (pol III transcript) and presented relative to the value for the TATA box of the *CYC1* promoter (lane 1). **C**. Northern blot analyses to examine the expression of the reporter (*VTC1*) or control genes (*RPS5* and *SCR1*) in WT or *taf1* strains carrying a reporter driven by the chimeric *CYC1*_*UAS*_+*AGP1*_*core*_ promoter in which the TATA box was substituted with TATATAAA (lanes 1–2, 5–6, 9–10, 13–14) or TA*GCGC*AA (lanes 3–4, 7–8, 11–12, 15–16) as indicated. Total RNA (20 μg) was isolated from these four strains (i.e., YTK17766/17769/17802/17804) grown to a logarithmic phase at 25°C in rich media containing 2% glucose (lanes 1, 3, 5, and 7) or 4% raffinose (lanes 9, 11, 13, and 15) (indicated as “25” above the blot), or further incubated at 37°C for 2 hours in each type of media (even-numbered lanes; indicated as “37” above the blot), blotted onto a membrane, and hybridized with the gene-specific probes indicated at the right. Autoradiogram hybridized with a *VTC1* probe and exposed for a longer time is also presented with an asterisk at the left. **D**. Raw data shown in **C** were quantified and presented graphically. Values for each transcript (*VTC1*/left graph, *RPS5*/right graph) derived from WT (black bars) or *taf1* (gray bars) strains were normalized against the level of *SCR1* (pol III transcript) and are presented relative to the value for the TATA box in the WT strain cultured in glucose-containing media at 25°C (i.e., the *VTC1/SCR1* or *RPS5/SCR1* values indicated to the left of lane 1).
(EPS)

S1 Table *Saccharomyces cerevisiae* strains used in this study. (DOC)

S2 Table Oligonucleotides used in this study. (DOC)

S3 Table PCR primers used for the construction of yeast strains in this study. (XLSX)
