# Supplementary material for: SAGA mediates transcription from the TATA-like element independently of Taf1p/TFIID but dependent on core promoter structures in Saccharomyces cerevisiae
Source: PLoS One. 2017 Nov 27;12(11):e0188435. doi: 10.1371/journal.pone.0188435 (PMC5703507; doi:10.1371/journal.pone.0188435)
Supplement: S1 Table — (DOC) [file pone.0188435.s007.doc]

S1 Table. *S. cerevisiae* strains used in this study (K. Watanabe et al.)

-----------------------------------------------------------------------------------------------------------------------------------------------------------------------------------------------------------

Strain Genotype Source

-----------------------------------------------------------------------------------------------------------------------------------------------------------------------------------------------------------

BY4741 *MATa his3Δ1 leu2Δ0 met15Δ0 ura3Δ0* Euroscarf

Y04228 *MATa his3Δ1 leu2Δ0 met15Δ0 ura3Δ0 Δspt3::kanMX4* Euroscarf

Y22.1 *MATα ura3-52 trp1-63 leu2-3,112 Δtaf1::hisG* pYN1/*TAF1* T. Kokubo (1998)

YTK2741 *MATα ura3-52 trp1-63 leu2-3,112 Δtaf1::hisG* pM1169/*TAF1* S. Takahata (2004)

YTK3778 *MATα ura3-52 trp1-63 leu2-3,112 Δtaf1::hisG* pM1746/*taf1-N568Δ* K. Watanabe (2015)

YTK11411 *MATa ura3Δ0 leu2Δ0 his3Δ1 met15Δ0 LYS2 Δtaf1::kanMX6* pYN1/*TAF1* Y. Ohyama (2010)

YTK11705 *MATα ura3Δ0 leu2Δ0 his3Δ1 MET15 lys2Δ0 Δtaf1::kanMX6* pM4770/*TAF1* Y. Ohyama (2010)

YTK11708 *MATα ura3Δ0 leu2Δ0 his3Δ1 MET15 lys2Δ0 Δtaf1::kanMX6* pM4773/*taf1-N568Δ* Y. Ohyama (2010)

YTK11871 *MATa ura3Δ0 leu2Δ0 his3Δ1 MET15 lys2Δ0 Δspt3::kanMX4 Δtaf1::kanMX6* pM4770/*TAF1* this study

YTK11873 *MATα ura3Δ0 leu2Δ0 his3Δ1 MET15 lys2Δ0 Δspt3::kanMX4 Δtaf1::kanMX6* pM4773/*taf1-N568Δ* this study

YTK13039 *MATa ura3Δ0 leu2Δ0 his3Δ1 MET15 lys2Δ0 Δspt3::kanMX4 Δtaf1::kanMX6* pYN1/*TAF1* this study

YTK16396 *MATα ura3-52 trp1-63 leu2-3,112 Δtaf1::hisG CYC1* promoter [#1, 3 & 4 mut]-*VTC1::LEU2* pM1169/*TAF1* K. Watanabe (2015)

YTK16397 *MATα ura3-52 trp1-63 leu2-3,112 Δtaf1::hisG CYC1* promoter [#1, 3 & 4 mut]-*VTC1::LEU2* pM1746/*taf1-N568Δ* K. Watanabe (2015)

YTK16398 *MATα ura3-52 trp1-63 leu2-3,112 Δtaf1::hisG CYC1* promoter [#1, 2, 3 & 4 mut]-*VTC1::LEU2* pM1169/*TAF1* K. Watanabe (2015)

YTK16399 *MATα ura3-52 trp1-63 leu2-3,112 Δtaf1::hisG CYC1* promoter [#1, 2, 3 & 4 mut]-*VTC1::LEU2* pM1746/*taf1-N568Δ* K. Watanabe (2015)

YTK16400 *MATα ura3-52 trp1-63 leu2-3,112 Δtaf1::hisG CYC1* promoter [#1, 3 & 4 mut + #2: TATTTAAA]-*VTC1::LEU2* pM1169/*TAF1* this study

YTK16401 *MATα ura3-52 trp1-63 leu2-3,112 Δtaf1::hisG CYC1* promoter [#1, 3 & 4 mut + #2: TATTTAAA]-*VTC1::LEU2* pM1746/*taf1-N568Δ* this study

YTK16402 *MATα ura3-52 trp1-63 leu2-3,112 Δtaf1::hisG CYC1* promoter [#1, 3 & 4 mut + #2: GTATTAAA]-*VTC1::LEU2* pM1169/*TAF1* this study

YTK16403 *MATα ura3-52 trp1-63 leu2-3,112 Δtaf1::hisG CYC1* promoter [#1, 3 & 4 mut + #2: GTATTAAA]-*VTC1::LEU2* pM1746/*taf1-N568Δ* this study

YTK16404 *MATα ura3-52 trp1-63 leu2-3,112 Δtaf1::hisG CYC1* promoter [#1, 3 & 4 mut + #2: TTTTTAAA]-*VTC1::LEU2* pM1169/*TAF1* this study

YTK16405 *MATα ura3-52 trp1-63 leu2-3,112 Δtaf1::hisG CYC1* promoter [#1, 3 & 4 mut + #2: TTTTTAAA]-*VTC1::LEU2* pM1746/*taf1-N568Δ* this study

YTK16406 *MATα ura3-52 trp1-63 leu2-3,112 Δtaf1::hisG CYC1* promoter [#1, 3 & 4 mut + #2: TACTTAAA]-*VTC1::LEU2* pM1169/*TAF1* this study

YTK16407 *MATα ura3-52 trp1-63 leu2-3,112 Δtaf1::hisG CYC1* promoter [#1, 3 & 4 mut + #2: TACTTAAA]-*VTC1::LEU2* pM1746/*taf1-N568Δ* this study

YTK16408 *MATα ura3-52 trp1-63 leu2-3,112 Δtaf1::hisG CYC1* promoter [#1, 3 & 4 mut + #2: TATATAGC]-*VTC1::LEU2* pM1169/*TAF1* this study

YTK16410 *MATα ura3-52 trp1-63 leu2-3,112 Δtaf1::hisG CYC1* promoter [#1, 3 & 4 mut + #2: TATATATC]-*VTC1::LEU2* pM1169/*TAF1* this study

YTK16412 *MATα ura3-52 trp1-63 leu2-3,112 Δtaf1::hisG CYC1* promoter [#1, 3 & 4 mut + #2: GCTATAAA]-*VTC1::LEU2* pM1169/*TAF1* this study

YTK16414 *MATα ura3-52 trp1-63 leu2-3,112 Δtaf1::hisG CYC1* promoter [#1, 3 & 4 mut + #2: TATATAAT]-*VTC1::LEU2* pM1169/*TAF1* this study

YTK16416 *MATα ura3-52 trp1-63 leu2-3,112 Δtaf1::hisG CYC1* promoter [#1, 3 & 4 mut + #2: CATTTAAA]-*VTC1::LEU2* pM1169/*TAF1* this study

YTK17558 *MATα ura3-52 trp1-63 leu2-3,112 Δtaf1::hisG CYC1* promoter [#1, 3 & 4 mut + #2: TATATACA]-*VTC1::LEU2* pM1169/*TAF1* this study

YTK17560 *MATα ura3-52 trp1-63 leu2-3,112 Δtaf1::hisG CYC1* promoter [#1, 3 & 4 mut + #2: TATATAGA]-*VTC1::LEU2* pM1169/*TAF1* this study

YTK17562 *MATα ura3-52 trp1-63 leu2-3,112 Δtaf1::hisG CYC1* promoter [#1, 3 & 4 mut + #2: TATATATA]-*VTC1::LEU2* pM1169/*TAF1* this study

YTK17564 *MATα ura3-52 trp1-63 leu2-3,112 Δtaf1::hisG CYC1* promoter [#1, 3 & 4 mut + #2: TATATAAC]-*VTC1::LEU2* pM1169/*TAF1* this study

YTK17566 *MATα ura3-52 trp1-63 leu2-3,112 Δtaf1::hisG CYC1* promoter [#1, 3 & 4 mut + #2: TATATAAG]-*VTC1::LEU2* pM1169/*TAF1* this study

YTK17568* *MATα ura3-52 trp1-63 leu2-3,112 Δtaf1::hisG CYC1* promoter [#1, 3 & 4 mut + #2: TATATAAT]-*VTC1::LEU2* pM1169/*TAF1* this study

YTK17570 *MATα ura3-52 trp1-63 leu2-3,112 Δtaf1::hisG CYC1* promoter [#1, 3 & 4 mut + #2: TATATACA]-*VTC1::LEU2* pM1746/*taf1-N568Δ* this study

YTK17572 *MATα ura3-52 trp1-63 leu2-3,112 Δtaf1::hisG CYC1* promoter [#1, 3 & 4 mut + #2: TATATAGA]-*VTC1::LEU2* pM1746/*taf1-N568Δ* this study

YTK17574 *MATα ura3-52 trp1-63 leu2-3,112 Δtaf1::hisG CYC1* promoter [#1, 3 & 4 mut + #2: TATATATA]-*VTC1::LEU2* pM1746/*taf1-N568Δ* this study

YTK17576 *MATα ura3-52 trp1-63 leu2-3,112 Δtaf1::hisG CYC1* promoter [#1, 3 & 4 mut + #2: TATATAAC]-*VTC1::LEU2* pM1746/*taf1-N568Δ* this study

YTK17578 *MATα ura3-52 trp1-63 leu2-3,112 Δtaf1::hisG CYC1* promoter [#1, 3 & 4 mut + #2: TATATAAG]-*VTC1::LEU2* pM1746/*taf1-N568Δ* this study

YTK17580 *MATα ura3-52 trp1-63 leu2-3,112 Δtaf1::hisG CYC1* promoter [#1, 3 & 4 mut + #2: TATATAAT]-*VTC1::LEU2* pM1746/*taf1-N568Δ* this study

YTK17582 *MATα ura3-52 trp1-63 leu2-3,112 Δtaf1::hisG AGP1* promoter [TATATACG]-*VTC1::LEU2* pM1169/*TAF1* this study

YTK17584 *MATα ura3-52 trp1-63 leu2-3,112 Δtaf1::hisG AGP1* promoter [TATATAGG]-*VTC1::LEU2* pM1169/*TAF1* this study

YTK17586 *MATα ura3-52 trp1-63 leu2-3,112 Δtaf1::hisG AGP1* promoter [TATATATG]-*VTC1::LEU2* pM1169/*TAF1* this study

YTK17590 *MATα ura3-52 trp1-63 leu2-3,112 Δtaf1::hisG AGP1* promoter [TATATAAG]-*VTC1::LEU2* pM1169/*TAF1* this study

YTK17591 *MATα ura3-52 trp1-63 leu2-3,112 Δtaf1::hisG AGP1* promoter [TATATAAC]-*VTC1::LEU2* pM1169/*TAF1* this study

YTK17593** *MATα ura3-52 trp1-63 leu2-3,112 Δtaf1::hisG AGP1* promoter [TATATAAT]-*VTC1::LEU2* pM1169/*TAF1* this study

YTK17595 *MATα ura3-52 trp1-63 leu2-3,112 Δtaf1::hisG AGP1* promoter [TATATACG]-*VTC1::LEU2* pM1746/*taf1-N568Δ* this study

YTK17597 *MATα ura3-52 trp1-63 leu2-3,112 Δtaf1::hisG AGP1* promoter [TATATAGG]-*VTC1::LEU2* pM1746/*taf1-N568Δ* this study

YTK17599 *MATα ura3-52 trp1-63 leu2-3,112 Δtaf1::hisG AGP1* promoter [TATATATG]-*VTC1::LEU2* pM1746/*taf1-N568Δ* this study

YTK17603 *MATα ura3-52 trp1-63 leu2-3,112 Δtaf1::hisG AGP1* promoter [TATATAAG]-*VTC1::LEU2* pM1746/*taf1-N568Δ* this study

YTK17604 *MATα ura3-52 trp1-63 leu2-3,112 Δtaf1::hisG AGP1* promoter [TATATAAC]-*VTC1::LEU2* pM1746/*taf1-N568Δ* this study

YTK17606 *MATα ura3-52 trp1-63 leu2-3,112 Δtaf1::hisG AGP1* promoter [TATATAAT]-*VTC1::LEU2* pM1746/*taf1-N568Δ* this study

YTK17748 *MATα ura3-52 trp1-63 leu2-3,112 Δtaf1::hisG AGP1_UAS_-CYC1_core_* [TATATAAA]-*VTC1::LEU2* pM1169/*TAF1* this study

YTK17750 *MATα ura3-52 trp1-63 leu2-3,112 Δtaf1::hisG AGP1_UAS_-CYC1_core_* [TAGCGCAA]-*VTC1::LEU2* pM1169/*TAF1* this study

YTK17766 *MATα ura3-52 trp1-63 leu2-3,112 Δtaf1::hisG CYC1_UAS_-AGP1_core_* [TATATAAA]-*VTC1::LEU2* pM1169/*TAF1* this study

YTK17769 *MATα ura3-52 trp1-63 leu2-3,112 Δtaf1::hisG CYC1_UAS_-AGP1_core_* [TAGCGCAA]-*VTC1::LEU2* pM1169/*TAF1* this study

YTK17784 *MATα ura3-52 trp1-63 leu2-3,112 Δtaf1::hisG AGP1_UAS_-CYC1_core_* [TATATAAA]-*VTC1::LEU2* pM1746/*taf1-N568Δ* this study

YTK17786 *MATα ura3-52 trp1-63 leu2-3,112 Δtaf1::hisG AGP1_UAS_-CYC1_core_* [TAGCGCAA]-*VTC1::LEU2* pM1746/*taf1-N568Δ* this study

YTK17802 *MATα ura3-52 trp1-63 leu2-3,112 Δtaf1::hisG CYC1_UAS_-AGP1_core_* [TATATAAA]-*VTC1::LEU2* pM1746/*taf1-N568Δ* this study

YTK17804 *MATα ura3-52 trp1-63 leu2-3,112 Δtaf1::hisG CYC1_UAS_-AGP1_core_* [TAGCGCAA]-*VTC1::LEU2* pM1746/*taf1-N568Δ* this study

YTK17952 *MATα ura3-52 trp1-63 leu2-3,112 Δtaf1::hisG AGP1* promoter [TATATAAA]-*VTC1::LEU2* pM1169/*TAF1* this study

YTK17954 *MATα ura3-52 trp1-63 leu2-3,112 Δtaf1::hisG AGP1* promoter [TAGCGCAA]-*VTC1::LEU2* pM1169/*TAF1* this study

YTK17956 *MATα ura3-52 trp1-63 leu2-3,112 Δtaf1::hisG AGP1* promoter [TATTTAAA]-*VTC1::LEU2* pM1169/*TAF1* this study

YTK17958 *MATα ura3-52 trp1-63 leu2-3,112 Δtaf1::hisG AGP1* promoter [GTATTAAA]-*VTC1::LEU2* pM1169/*TAF1* this study

YTK17960 *MATα ura3-52 trp1-63 leu2-3,112 Δtaf1::hisG AGP1* promoter [TTTTTAAA]-*VTC1::LEU2* pM1169/*TAF1* this study

YTK17962 *MATα ura3-52 trp1-63 leu2-3,112 Δtaf1::hisG AGP1* promoter [TACTTAAA]-*VTC1::LEU2* pM1169/*TAF1* this study

YTK17964 *MATα ura3-52 trp1-63 leu2-3,112 Δtaf1::hisG AGP1* promoter [TATATAGC]-*VTC1::LEU2* pM1169/*TAF1* this study

YTK17966 *MATα ura3-52 trp1-63 leu2-3,112 Δtaf1::hisG AGP1* promoter [TATATATC]-*VTC1::LEU2* pM1169/*TAF1* this study

YTK17968 *MATα ura3-52 trp1-63 leu2-3,112 Δtaf1::hisG AGP1* promoter [GCTATAAA]-*VTC1::LEU2* pM1169/*TAF1* this study

YTK17970 *MATα ura3-52 trp1-63 leu2-3,112 Δtaf1::hisG AGP1* promoter [TATATAAT]-*VTC1::LEU2* pM1169/*TAF1* this study

YTK17972 *MATα ura3-52 trp1-63 leu2-3,112 Δtaf1::hisG AGP1* promoter [CATTTAAA]-*VTC1::LEU2* pM1169/*TAF1* this study

YTK17974 *MATα ura3-52 trp1-63 leu2-3,112 Δtaf1::hisG AGP1* promoter [TATATAAA]-*VTC1::LEU2* pM1746/*taf1-N568Δ* this study

YTK17976 *MATα ura3-52 trp1-63 leu2-3,112 Δtaf1::hisG AGP1* promoter [TAGCGCAA]-*VTC1::LEU2* pM1746/*taf1-N568Δ* this study

YTK17978 *MATα ura3-52 trp1-63 leu2-3,112 Δtaf1::hisG AGP1* promoter [TATTTAAA]-*VTC1::LEU2* pM1746/*taf1-N568Δ* this study

YTK17980 *MATα ura3-52 trp1-63 leu2-3,112 Δtaf1::hisG AGP1* promoter [GTATTAAA]-*VTC1::LEU2* pM1746/*taf1-N568Δ* this study

YTK17982 *MATα ura3-52 trp1-63 leu2-3,112 Δtaf1::hisG AGP1* promoter [TTTTTAAA]-*VTC1::LEU2* pM1746/*taf1-N568Δ* this study

YTK17984 *MATα ura3-52 trp1-63 leu2-3,112 Δtaf1::hisG AGP1* promoter [TACTTAAA]-*VTC1::LEU2* pM1746/*taf1-N568Δ* this study

YTK18955 *MATa ura3Δ0 leu2Δ0 his3Δ1 met15Δ0 LYS2 Δtaf1::kanMX6 AGP1* promoter [TATATAAA]-*VTC1::LEU2* pYN1/*TAF1* this study

YTK18964 *MATa ura3Δ0 leu2Δ0 his3Δ1 MET15 lys2Δ0 Δspt3::kanMX4 Δtaf1::kanMX6 AGP1* promoter [TATATAAA]-*VTC1::LEU2* pYN1/*TAF1* this study

YTK18974 *MATa ura3Δ0 leu2Δ0 his3Δ1 met15Δ0 LYS2 Δtaf1::kanMX6 AGP1* promoter [TATATAAA]-*VTC1::LEU2* pM4770/*TAF1* this study

YTK18986 *MATa ura3Δ0 leu2Δ0 his3Δ1 met15Δ0 LYS2 Δtaf1::kanMX6 AGP1* promoter [TATATAAA]-*VTC1::LEU2* pM4773/*taf1-N568Δ* this study

YTK18998 *MATa ura3Δ0 leu2Δ0 his3Δ1 MET15 lys2Δ0 Δspt3::kanMX4 Δtaf1::kanMX6 AGP1* promoter [TATATAAA]-*VTC1::LEU2* pM4770/*TAF1* this study

YTK19010 *MATa ura3Δ0 leu2Δ0 his3Δ1 MET15 lys2Δ0 Δspt3::kanMX4 Δtaf1::kanMX6 AGP1* promoter [TATATAAA]-*VTC1::LEU2* pM4773/*taf1-N568Δ* this study

-----------------------------------------------------------------------------------------------------------------------------------------------------------------------------------------------------------

YTK17568* and YTK17593** are genetically the same as YTK16414 and YTK17970, respectively, although the methods of constructions were different (see details in the Materials and Methods section).
