# Supplementary material for: SAGA mediates transcription from the TATA-like element independently of Taf1p/TFIID but dependent on core promoter structures in Saccharomyces cerevisiae
Source: PLoS One. 2017 Nov 27;12(11):e0188435. doi: 10.1371/journal.pone.0188435 (PMC5703507; doi:10.1371/journal.pone.0188435)
Supplement: S2 Table — (DOC) [file pone.0188435.s008.doc]

S2 Table Oligonucleotides used in this study (K. Watanabe et al.)

--------------------------------------------------------------------------------------------------------------------------------------------------------------

ID Sequence

--------------------------------------------------------------------------------------------------------------------------------------------------------------

TK493 5'-ATGTCTGACACCGAAGCTCCA-3'

TK494 5'-TTAACGGTTAGACTTGGCAAC-3'

TK2496 5'-AAATAGATATTAGCACGTGTCTCGG-3'

TK4283 5'-TTTGTGCGTAACCCACGCTTACGATATTGGAATTACAATT-3'

TK6582 5'-GTCGACGGATCCCCGGGAATTCGATCCGCAGGCTAACCGGAA-3'

TK7872 5'-TCGGATAGAATGGCAAGAGC-3'

TK7873 5'-TCTGGCGTAGTGTTTAATCG-3'

TK7875 5'-AAAAAGCACAACAAAGTGGG-3'

TK8195 5'-ATGTCGTCGTCGAAGTCTCT-3'

TK8196 5'-TACCCGTACCCAACGCAATC-3'

TK8260 5'-CGGTATCCGCTAATAGAGAG-3'

TK8267 5'-GAATTCCCGGGGATCCGTCGACGATGACTTTACCTAGCACTC-3'

TK8268 5'-TTTTCCCAGGTGTTCTTTGTAATAATGGTGCTGAAGACATTGTGCGAAGCTATCTTTGTC-3'

TK8678 5'-GTATGTAAAACAAATGAGTA-3'

TK9013 5'-TAACTTAGTGTTAGCGTCATT-3'

TK9030 5'-ATGTCTTCAGCACCATTATTACAA-3'

TK9171 5'-TCATTGTACCCATGGCAACA-3'

TK9507 5'-AGGCTGTAATGGCTTTCT-3'

TK9727 5'-ATGACTGAATTCAAGGCCGGTTCTGCTAAG-3'

TK9745 5'-TTACTCACAGGCTTTTTTCAAGTAGGTAAT-3'

TK10036 5'-GAATTCCCGGGGATCCGTCGACTGAATCTAAAATTCCCGGGA-3'

TK10081 5'-CCACAATGTGCGAGTAAATCC-3'

TK10257 5'-CATACAGAGCACATGCATGCC-3'

TK10267 5'-GCAGAGGAGAGTTATCACTCCTTCCATCCT-3'

TK10408 5'-TAAACCTACACCAAGGCCTC-3'

TK12262 5'-ATACGATTAAACACTACGCCAGATTTCCACAATGAGCTCGCTGTGAAGATCCC-3'

TK12517 5'-CGTAATACTCATTTGTTTTACATACATATATAAATGCTTTGTCTTTGATGGT-3'

TK12518 5'-CGTAATACTCATTTGTTTTACATACATAGCGCAATGCTTTGTCTTTGATGGT-3'

TK12519 5'-CGTAATACTCATTTGTTTTACATACATATTTAAATGCTTTGTCTTTGATGGT-3'

TK12520 5'-CGTAATACTCATTTGTTTTACATACAGTATTAAATGCTTTGTCTTTGATGGT-3'

TK12521 5'-CGTAATACTCATTTGTTTTACATACATTTTTAAATGCTTTGTCTTTGATGGT-3'

TK12522 5'-CGTAATACTCATTTGTTTTACATACATACTTAAATGCTTTGTCTTTGATGGT-3'

TK12524 5'-CGTAATACTCATTTGTTTTACATACATATATATCTGCTTTGTCTTTGATGGT-3'

TK12525 5'-CGTAATACTCATTTGTTTTACATACAGCTATAAATGCTTTGTCTTTGATGGT-3'

TK12526 5'-CGTAATACTCATTTGTTTTACATACATATATAATTGCTTTGTCTTTGATGGT-3'

TK12527 5'-CGTAATACTCATTTGTTTTACATACACATTTAAATGCTTTGTCTTTGATGGT-3'

TK12873 5'-GATCCACTAGTTCTAGAGCGGC-3'

TK12918 5'-CGACGACACATGATCATATGGCATGCATGTGCTCTGTATGTATATACAACTCTTGTTTTCTTCTTTTCTCTAA-3'

TK12919 5'-CGACGACACATGATCATATGGCATGCATGTGCTCTGTATGTATATAGAACTCTTGTTTTCTTCTTTTCTCTAA-3'

TK12920 5'-CGACGACACATGATCATATGGCATGCATGTGCTCTGTATGTATATATAACTCTTGTTTTCTTCTTTTCTCTAA-3'

TK12921 5'-CGACGACACATGATCATATGGCATGCATGTGCTCTGTATGTATATAACACTCTTGTTTTCTTCTTTTCTCTAA-3'

TK12922 5'-CGACGACACATGATCATATGGCATGCATGTGCTCTGTATGTATATAAGACTCTTGTTTTCTTCTTTTCTCTAA-3'

TK12923 5'-CGACGACACATGATCATATGGCATGCATGTGCTCTGTATGTATATAATACTCTTGTTTTCTTCTTTTCTCTAA-3'

TK12924 5'-TTTGTTATTTTCCTCGTAATACTCATTTGTTTTACATACATATATACGTGCTTTGTCTTTGATGGTCTG-3'

TK12925 5'-TTTGTTATTTTCCTCGTAATACTCATTTGTTTTACATACATATATAGGTGCTTTGTCTTTGATGGTCTG-3'

TK12926 5'-TTTGTTATTTTCCTCGTAATACTCATTTGTTTTACATACATATATATGTGCTTTGTCTTTGATGGTCTG-3'

TK12928 5'-TTTGTTATTTTCCTCGTAATACTCATTTGTTTTACATACATATATAACTGCTTTGTCTTTGATGGTCTG-3'

TK12929 5'-TTTGTTATTTTCCTCGTAATACTCATTTGTTTTACATACATATATAATTGCTTTGTCTTTGATGGTCTG-3'

TK13002 5'-TTTGTTATTTTCCTCGTAATACTCATTTGTTTTACATACATATATAGCTGCTTTGTCTTTGATGGTCTG-3'

TK13577 5'-TGCAGGAATTCGATATCAAGCT-3'

TK13578 5'-TAGAACTAGTGGATCTCGGATAGAATGGCAAGAGC-3'

TK13579 5'-TATCGAATTCCTGCACAAGAGCTTAACTCTACGGC-3'

TK13612 5'-AAGAAATGGCTAGAAGGCACT-3'

TK13614 5'-GCAGATCCGCCAGGCCCAGCTTGGCAGTGGTTATC-3'

TK13618 5'-GCCTGGCGGATCTGCTCG-3'

TK13619 5'-TTCTAGCCATTTCTTGTGTATATATAGCGTGGATGG-3'

--------------------------------------------------------------------------------------------------------------------------------------------------------------
